# The Molecular Profiles of Neural Stem Cell Niche in the Adult Subventricular Zone

**DOI:** 10.1371/journal.pone.0050501

**Published:** 2012-11-29

**Authors:** Cheol Lee, Jingqiong Hu, Sherry Ralls, Toshio Kitamura, Y. Peng Loh, Yanqin Yang, Yoh-suke Mukouyama, Sohyun Ahn

**Affiliations:** 1 Program in Genomics of Differentiation, *Eunice Kennedy Shriver* National Institute of Child Health and Human Development, National Institutes of Health, Bethesda, Maryland, United States of America; 2 Laboratory of Stem Cell and Neuro-Vascular Biology, Genetics and Developmental Biology Center, National Heart, Lung, and Blood Institute, National Institutes of Health, Bethesda, Maryland, United States of America; 3 Division of Cellular Therapy, Advanced Clinical Research Center, The Institute of Medical Science, The University of Tokyo, Tokyo, Japan; 4 Section on Cellular Neurobiology, Program on Developmental Neuroscience, *Eunice Kennedy Shriver* National Institute of Child Health and Human Development, National Institutes of Health, Bethesda, Maryland, United States of America; 5 DNA Sequencing and Genomics Core Facility, National Heart, Lung, and Blood Institute, National Institutes of Health, Bethesda, Maryland, United States of America; Universitat Pompeu Fabra, Spain

## Abstract

Neural stem cells (NSCs) reside in a unique microenvironment called the neurogenic niche and generate functional new neurons. The neurogenic niche contains several distinct types of cells and interacts with the NSCs in the subventricular zone (SVZ) of the lateral ventricle. While several molecules produced by the niche cells have been identified to regulate adult neurogenesis, a systematic profiling of autocrine/paracrine signaling molecules in the neurogenic regions involved in maintenance, self-renewal, proliferation, and differentiation of NSCs has not been done. We took advantage of the genetic inducible fate mapping system (GIFM) and transgenic mice to isolate the SVZ niche cells including NSCs, transit-amplifying progenitors (TAPs), astrocytes, ependymal cells, and vascular endothelial cells. From the isolated cells and microdissected choroid plexus, we obtained the secretory molecule expression profiling (SMEP) of each cell type using the Signal Sequence Trap method. We identified a total of 151 genes encoding secretory or membrane proteins. In addition, we obtained the potential SMEP of NSCs using cDNA microarray technology. Through the combination of multiple screening approaches, we identified a number of candidate genes with a potential relevance for regulating the NSC behaviors, which provide new insight into the nature of neurogenic niche signals.

## Introduction

In the postnatal mammalian brain, neural stem cells (NSCs) are retained in a unique compartment after embryonic development and generate new cells throughout the life of an animal. Under the normal condition, postnatal neurogenesis occurs only in two major neurogenic regions, the subventricular zone (SVZ) of the lateral ventricle and the subgranular zone (SGZ) of the dentate gyrus of the hippocampus [1]–[3]. While the cells in the non-neurogenic regions do not produce new cells *in vivo*, they can become neurogenic under proper culture conditions *in vitro*, indicating the importance of the permissive environment in which NSCs reside. Such a specialized microenvironment is called the NSC niche and provides the appropriate cues that regulate NSC behaviors, such as maintenance, self-renewal, proliferation, and generation of progenies [4], [5]. The importance of the NSC niche in regulating NSC behaviors has been shown by transplantation studies. The neural progenitors grafted into the homotopic or heterotopic neurogenic regions produced new neurons whereas those grafted into the non-neurogenic region did not [6], [7]. These studies further suggest that certain signaling cues in the NSC niche govern the neurogenic potential of NSCs. However, the nature of niche signals is not fully elucidated.

The NSC niche is composed of cellular components and extracellular substrates, which collectively provide residing milieu for NSCs and regulate NSC behaviors [8]–[10]. The cellular components of the SVZ niche are NSCs as well as their progenies and other differentiated neighboring cells. NSCs (type B1 and B2 cells) are slowly dividing progenitors possessing self-renewal capacity and astrocyte-like features [11]. NSCs generate actively dividing transit-amplifying progenitors (TAPs, also known as type C cells), which in turn produce neuroblasts (type A cells) that differentiate into interneurons and eventually incorporate into the olfactory bulb circuitry [11], [12]. Ependymal cells are single-layered epithelial cells lining the wall of the lateral ventricle, and thereby act as the brain-CSF (cerebrospinal fluid) interface [13]. Ependymal cells are often closely associated with type B1 cells to form a pinwheel-like structure, where the ependymal cells surround the apical processes of type B1 cells [14].

The vasculature is a well-known NSC niche component as NSCs and TAPs are located in close proximity to the blood vessels [15], [16]. Endothelial cells also secrete growth factors that regulate NSC behaviors [17], [18]. Astrocytes, the most abundant cell type in the adult mammalian brain, provide structural, metabolic, and trophic support for neurons and modulate synaptic transmission. Astrocytes can also support proliferation of adult SVZ NSCs/TAPs and differentiation into neuroblasts *in vitro* [19]. Recently, the NSCs (type B1 cells) were shown to contain the primary cilium, which acts as a sensor for various signals including Hedgehog and Wnt [14], [20], [21]. Since the primary cilium of the NSCs protrudes into the lumen of the lateral ventricle, it is likely that the CSF may also contain important signals to control NSC behaviors. As the choroid plexus (CP) in the posterior lateral ventricle is the primary source of the CSF [22], the epithelial cells of CP can also be considered as a cellular component of the SVZ niche.

Because of the well-organized cytoarchitecture and NSC’s direct contact to the vasculature and CSF, it has been thought that niche cells influence NSC behaviors by cell-cell contact and/or by releasing secretory signaling molecules. Indeed, many studies have shown that various morphogens, growth factors, extracellular substrates, and neurotransmitters are involved in NSC proliferation, lineage determination, and progeny’s functional maturation. For example, known signaling molecules including Sonic hedgehog (Shh), Wnt, and BMP are responsible for regulating NSC behaviors [23]–[26]. Various growth factors such as PEDF, EGF, FGF2, and BDNF as well as extracellular matrix components, such as Tenascin-C, have also been demonstrated as niche signals [17], [27]–[31]. In an attempt to search for autocrine/paracrine factors produced by niche cells, several studies examined the conditioned medium of neurosphere cultures by mass spectrometry and identified molecules such as Cystatin C, Apolipoprotein E, and DSD-1-proteoglycan [32], [33]. Another recent study utilized a co-culture system of a human NSC line and human umbilical cord endothelial cells to investigate the communication between endothelial cells and NSCs via soluble factors [34]. However, there has not been any report that profiled the signaling molecules in the neurogenic niche in a systematic and comprehensive manner.

In order to gain a better understanding of how NSCs communicate with surrounding niche cells, we aim to provide a systematic profiling of signaling molecules in the SVZ neurogenic niche in a cell-type specific manner in this study. First, for the systematic exploration of autocrine/paracrine as well as cell-cell contact-based signaling factors in the SVZ niche, we used the Signal Sequence Trap by Retrovirus-mediated Expression screening (SST-REX) method [35]. SST-REX has successfully identified secretory or cell surface molecules in various tissues and cell types including hematopoietic stem cells [36], vascular smooth muscle cells [37], cardiac myocytes [38], and the hippocampal neural stem/progenitor cells [39]. SST-REX is a cloning strategy for selecting or screening the cDNA fragments encoding the N-terminal signal sequences, which are common in cDNAs of the secreted or type I transmembrane proteins [35]. In this method, cDNA library fragments are inserted into the pMX-SST library vector, which contains the N-terminal truncated and constitutively active mutant form of myeloproliferative leukemia virus oncogene (ΔMPL^M^) [35], [40]. MPL is a member of the hematopoietic cytokine receptor family and known to transmit a proliferative signal in interleukin-3 (IL-3)-dependent cell lines including the mouse pro-B cell line Ba/F3 [41]. Since ΔMPL^M^ lacks a signal sequence, only a chimeric receptor consisting of the signal sequence derived from the inserted cDNA and the ΔMPL^M^ can be directed to the cell membrane and thereby allow cells to proliferate. After the surviving clones are selected, the genomic DNA of each clone is sequenced for the integrated cDNA. In this study, we identified the secretory and cell surface molecules from the 6 cellular components of the SVZ niche, which are NSCs, TAPs, astrocytes, ependymal cells, vascular endothelial cells, and the choroid plexus. We found SVZ niche molecules with the identified function or potential relevance for regulating the NSC behaviors as niche signals. Together, our niche cell isolation strategy and the secretory molecule screening process provide new insight into the nature of NSC niche signals.

## Materials and Methods

### Animals

*Gli1^CreER/+^* mice [42] were crossed with *hGFAP-GFP* mice [43] to generate *Gli1^CreER/+^;hGFAP-GFP* mice, which were then crossed with *R26^tdTomato/tdTomato^* mice [44] to generate *Gli1^CreER/+^;hGFAP-GFP;R26^tdTomato/+^*. *FoxJ1-Cre* mice [45] were crossed with *R26^YFP/YFP^* to generate *FoxJ1-Cre;R26^YFP/+^*. *Tie2-GFP* mice [46] were purchased from Jackson Laboratory (Jax mouse strain Tg(TIE2GFP)287Sato/J). *Nestin-Cre* mice [47] were crossed with *R26^tdTomato/tdTomato^* mice to generate *Nestin-Cre;R26^tdTomato/+^* mice. 2–3 month-old male and female mice were used for signal sequence trap screening, and only male mice were used for cDNA microarray. To induce transgene expression, 6–8 week-old *Gli1^CreER/+^;hGFAP-GFP;R26^tdTomato/+^* mice received tamoxifen (10 mg) orally for 2 consecutive days and were sacrificed 3 weeks later. *Tie2-GFP* mice were backcrossed to C57BL/6 mice for at least 10 generations and maintained in C57BL/6 background. All the other mouse lines were maintained on an outbred Swiss Webster background. All animals used in this study were handled according to protocols approved by the Institutional Animal Care and Use Committee of the National Institutes of Health.

### Materials

1-oleoyl-LPC (18∶1) was purchased from Avanti Polar Lipid Inc. Human plasma transthyretin was purchased from Calbiochem. Recombinant mouse Enpp2 and mouse Sparcl1 were purchased from R&D Systems. Mouse CPE was expressed and purified under contract by Creative Biolabs.

### Fluorescence-activated Cell Sorting (FACS)-isolation of NSC Niche Cells

The SVZ of *Gli1^CreER/+^;hGFAP-GFP;R26^tdTomato/+^* mice 3 weeks after tamoxifen treatment or *FoxJ1-Cre;R26^YFP/+^* mice were microdissected and dissociated using papain (Worthington Biochemical Corp). The cell suspension was triturated and filtered through a 40 µm cell strainer and myelin components were removed by Myelin Removal Beads (Miltenyi Biotec). The SVZ of *Tie2-GFP* brains were processed according to published protocol [46]. 10–12 mice were used for each experiment. NSCs, TAPs, and astrocytes were isolated based on their expression of *GFAP* (GFP+) and/or *Gli1* (tdTomato+) using a MoFlo cell sorter (Beckman Coulter). Ependymal cells and endothelial cells were isolated based on *FoxJ1* (YFP+) and *Tie2* (GFP+) expression, respectively. Gates were set using *hGFAP-GFP* mice for GFP or YFP control, *Nestin-Cre;R26^tdTomato/+^* mice for tdTomato control, and wild-type mice as a negative control. Dead cells were excluded by 7-AAD (Invitrogen) staining.

### Cell Culture

The Plat-E virus producing cell line [48] was cultured in DMEM containing 10% fetal bovine serum (FBS), 1 µg/mL puromycin, and 10 µg/mL blasticidin. A murine IL-3 dependent pro-B cell line Ba/F3 cells [49] were cultured in RPMI media containing 10% FBS and 10 ng/ml IL-3 (Gibco). FACS-isolated GFP+/tdTomato+ cells were cultured as neurospheres in DMEM/F-12 medium (Invitrogen) with 20 ng/ml EGF (R&D Systems), FGF2 (R&D Systems), and B-27 supplement (Invitrogen). Neurospheres were passaged by Accutase dissociation (Invitrogen).

### Construction of the cDNA Library for SST-REX

SST-REX) was performed as described previously [35]. Total RNA was extracted from dissected choroid plexus or FACS-isolated cells and amplified by MessageAmp II aRNA Amplification kit (Ambion). cDNA was synthesized and size fractionated by SuperScript Choice System (Invitrogen) based on the manufacturer’s instructions. The size selected (>500 bp) cDNA fractions were ligated into the *BstXI* site of pMX-SST cloning vector using *BstXI* adapters and introduced into DH10B cells (Invitrogen) using Gene Pulser II (BioRad). Transformed cells were cultured overnight and plasmid DNA was prepared using a HiSpeed Plasmid Maxi Kit (Qiagen).

### SST-REX

The library plasmid and pMX-GFP control plasmid were transfected into Plat-E cells using either the FuGENE6 (Roche) or calcium phosphate transfection system. Retroviral supernatant was harvested 48 hr after transfection and Ba/F3 cells were infected as described [50]. 24 hr later, the cells were washed three times with PBS and seeded in twelve 96-multiwell plates in the absence of IL-3 with 10^4^ cells/well density. After 2-week culture, genomic DNAs were extracted from IL-3-independent Ba/F3 clones, followed by PCR to verify the integrated cDNAs using signal sequence primers (GGGGGTGGACCATCCTCTA and CGCGCAGCTGTAAACGGTAG) [35]. Finally, the resulting PCR fragments were sequenced and analyzed by BLAST search.

### Microarray Processing and Hybridization

The GFP+/tdTomato+ cells were collected from the SVZ or cerebella of *Gli1^CreER/+^;hGFAP-GFP;R26^tdTomato/+^* mice by FACS and RNAs were extracted. Approximately 50 ng RNA samples were amplified using the WT-Ovation™ Pico RNA Amplification System (NuGEN) in a 3-step process as recommended by the manufacturer. All processes were preformed in an automated manner using the GeneChip Array Station. Following purification and quantitation, 5 µg of the amplified cDNA was fragmented and labeled with the FL-Ovation™ cDNA Biotin Module using a proprietary two-step fragmentation and labeling process. Biotinylated cDNA was hybridized to Affymetrix mouse genome 430 2.0 gene chips at 45°C overnight, followed by washing and staining using FS450 fluidics station. Hybridization, washing and laser scanning of Mouse Genome 430 2.0 arrays were performed according to the manufacturer’s protocol (Affymetrix). Arrays were scanned by the 7G GCS3000 Scanner.

### Microarray Data Analysis

The raw microarray datasets were analyzed by the R-Project Bioconductor affy package (www.bioconductor.org). The probeset expression values were generated for each microarray chip using Robust Multichip Analysis for background adjustment, quantile normalization, and median polish summarization. The quantile normalization forces all microarray chips to conform to the same distribution of 45,101 probesets. Normalized datasets were applied for Principal Component Analysis and detected two outliers among 16 chips. After removing the outliers, the data were subjected to significant differential expression genes search using Bioconductor ‘limma’ package, which implements moderate t-statistics with the Benjamin-Hochberg multiple testing correction. The identified genes were obtained by the filters of greater than 4-fold changes with less than 1% false discovery rate (FDR).

### Tissue Processing and Immunohistochemistry

2–3 month-old mice were deeply anesthetized with Nembutal and transcardially perfused with 4% paraformaldehyde (PFA). The dissected brains were fixed at 4°C overnight, cryoprotected in 30% sucrose/PBS, embedded in OCT and cryosectioned at 20 µm thickness. Fluorescent immunohistochemistry was performed essentially as described in Ahn and Joyner, 2005. The primary antibodies used and their dilution factors are as follows: Rabbit IgG against S100β (1∶500, Sigma), Sox2 (1∶500, Chemicon), GFP (1∶500, Invitrogen), and GFAP (1∶1000, DakoCytomation); Mouse IgG against GFAP (1∶500, Chemicon), Nestin (1∶100, Chemicon), TuJ1 (1∶500, Chemicon), O4 (1∶500, Chemicon), Mash1 (1∶500, BD Pharmingen), and CNPase (1∶500, Chemicon); rat IgG against GFP (1∶500, Nacalai USA, Inc.), CD31 (1∶200, BD Pharmingen) and CD24 (1∶200, BD Pharmingen). The cell type-specific expression of marker genes is listed in Table S1. FACS isolated cells were transferred to poly-L-lysine (or fibronectin) coated chamber slides (Millipore) and spun down to attach cells on the slide surface. Cells were fixed with 4% PFA for 10 min and processed for immunocytochemistry using the same method used for tissue staining.

### Quantitative Real-time PCR

cDNA of FACS-isolated niche cells (NSCs, TAPs, astrocytes, ependymal cells, endothelial cells) and the choroid plexus were prepared by sequence-specific reverse transcription and amplification [51]. Primers (DELTAgene Assays, Fluidigm) were combined to a final concentration 200 nM. 3 ng of total RNA extracted from the choroid plexus or 100 each of FACS-isolated niche cells were collected directly into 5 µl of CellsDirect 2×Reaction Mix (CellsDirect qRT-PCR kit, Invitrogen). The prepared samples were immediately frozen and stored at −80°C until use. Each frozen sample was thawed and added 4 µl of a solution containing a mixture of RT/Taq enzyme (CellsDirect qRT-PCR kit, Invitrogen), combined primers, and nuclease-free water. Cell lysis and reverse transcription were performed at 50°C for 15 min followed by reverse transcriptase inactivation at 95°C for 2 min. In the same tube, the specific target amplification was performed by denaturing at 95°C for 15 sec, and annealing/amplification at 60°C for 4 min for 18 cycles. These preamplified products were diluted 200-fold and used for qRT-PCR analysis measuring SYBR Green incorporation (LightCycler 480 SYBR Green I Master, Roche) in a Roche Light Cycler 480 PCR machine. *Gapdh* was used as a reference gene. The same primers used for the reverse transcription and specific target amplification were used for qRT-PCR and the sequence of primers are listed in Table S2.

### *In vitro* Functional Validation

*Nestin-Cre;R26^tdTomato/+^* mice were used for the computer-based assessment of the neurosphere size and number. The SVZ was dissected, dissociated (papain, Worthington Biochemical Corp), and myelin was removed (Miltenyi Biotec). Cells were plated and cultured under the same conditions as described above. tdTomato expressing primary neurospheres were collected, dissociated (Accutase, Invitrogen) and replated at a clonal density (10 cells/µl in 0.1 ml of media) [52] in flat-bottom 96-well plates, and cultured with vehicle or each reagent (LPC: 10 µM, Enpp2∶100 ng/ml, Ttr: 50 µg/ml, Sparcl1∶30 ng/ml, and CPE: 100 nM) for 6–7 days *in vitro*. Only neurospheres greater than 100 µm in diameter were quantified. The total number of cells was estimated using a XTT cell proliferation assay kit according to the manufacturer’s instructions (ATCC). Following neurosphere assay, XTT assay solution was added to neurosphere culture medium and incubated at 37°C for 4 hr. Absorbance values were obtained at 450 nm in a VICTOR3 plate reader (Perkin-Elmer).

For neural differentiation, SVZ-derived neurospheres were dissociated and plated at 5,000 cells/well in 96-well poly-L-lysine (Sigma) coated plates in DMEM/F12 medium containing 1% FBS and B27. Cells were cultured with either vehicle or each reagent for 5 days. For qRT-PCR analysis, total mRNA was extracted from differentiation-induced cells using the RNeasy MinElute kit (Qiagen). cDNA was synthesized using an iScript kit (Biorad). qRT-PCR was performed as described above and the sequence of primers are listed in Table S2.

### Image Analysis and Quantification

The images were captured using a Zeiss LSM 510 confocal microscope system or a Leica DMI4000B inverted fluorescence microscope equipped with a DFC340 FX digital camera for fluorescent images. The image acquisition and processing were done with either LSM 5 (Zeiss) or Volocity (Perkin-Elmer) and Adobe Creative Suite 3 Photoshop softwares. The size and number of neurospheres were measured using the Volocity measurement function (Perkin-Elmer).

### Statistical Analysis

A two-tailed paired t-test was used to decide the statistical significance for the *in vitro* functional validation experiments. Results are presented in a scatter plot, with bars representing the mean. Significance was set at p<0.05.

## Results

### Isolation of NSC niche cells

**Figure 1:**
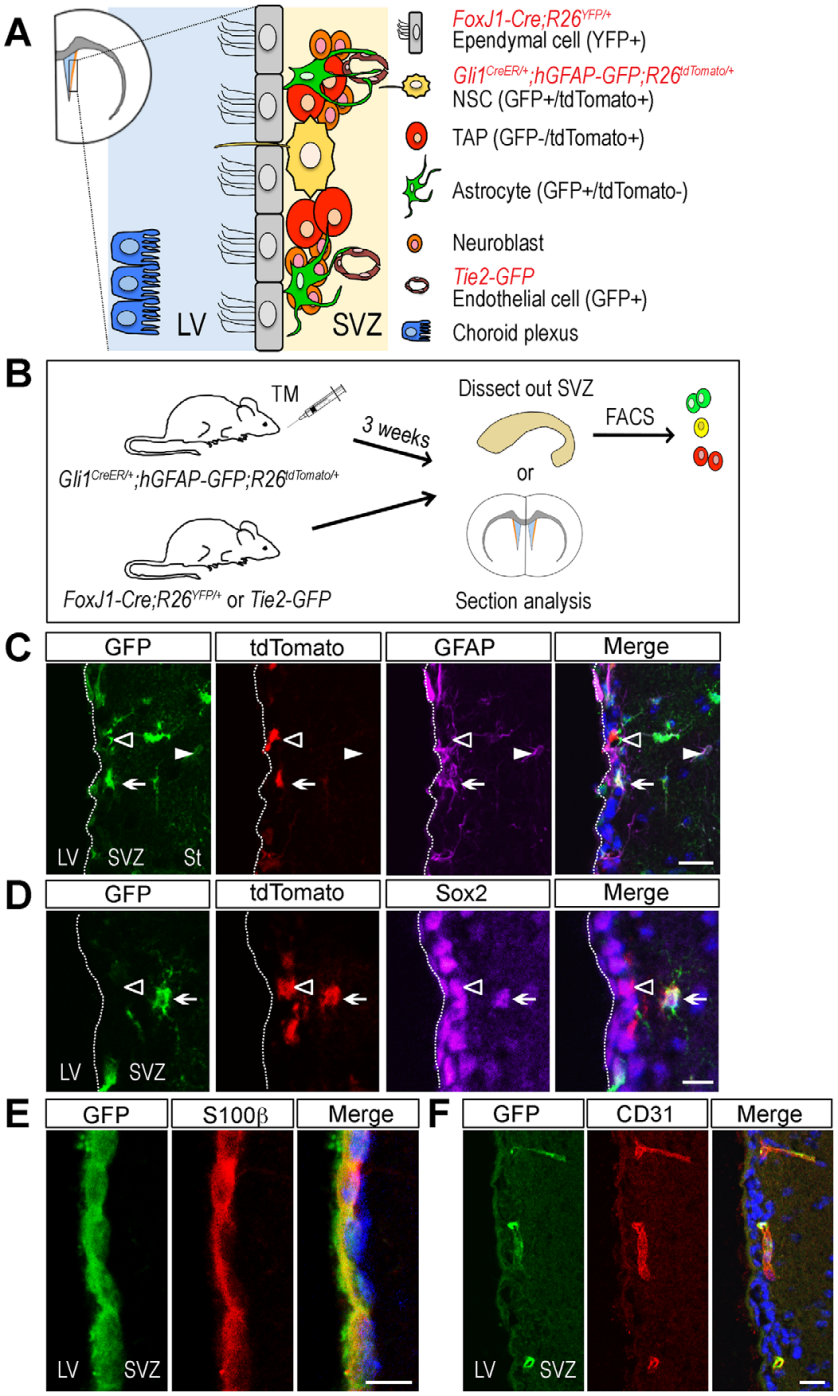
Transgenic markers are expressed by specific NSC niche cell types. (A) A schematic of the adult mouse forebrain in the coronal plane and cellular components of the neural stem cell (NSC) niche. Ependymal cells in *FoxJ1-Cre;R26^YFP/+^* mice express YFP. NSCs, transit-amplifying progenitors (TAPs), and astrocytes in *Gli1^CreER/+^;hGFAP-GFP;R26^tdTomato/+^* mice express GFP/tdTomato, only tdTomato, and only GFP, respectively. Vascular endothelial cells in *Tie2-GFP* mice express GFP. LV: lateral ventricle, SVZ: subventricular zone. (B) A schema represents the induction of transgene in *Gli1^CreER/+^;hGFAP-GFP;R26^tdTomato/+^* mice by tamoxifen treatment. Three weeks after the treatment, SVZs were dissected, dissociated, and subjected to FACS or analyzed for the *in vivo* transgene expression. *FoxJ1-Cre;R26^YFP/+^* or *Tie2-GFP* mice were also used for FACS or the *in vivo* transgene expression analysis. (C and D) A coronal section of the SVZ of *Gli1^CreER/+^;hGFAP-GFP;R26^tdTomato/+^* mouse. *GFP* expressing cells (green) are localized in the SVZ as well as in the striatum (St). *Gli1* lineage cells (tdTomato+, red) are located predominantly in the SVZ. Dashed line indicates the border between the lumen of the LV and the SVZ. (C) Immunofluorescent staining for GFAP (magenta) labels NSCs (GFP+/tdTomato+, arrow) and astrocytes (GFP+/tdTomato−, arrow head) in the striatum. TAPs (GFP−/tdTomato+, open arrow head) are not stained by GFAP. (D) Immunofluorescent staining for Sox2 (magenta) labels NSCs (GFP+/tdTomato+, arrow) and TAPs (GFP−/tdTomato+, open arrow head). (E) A coronal section of the SVZ of *FoxJ1-Cre;R26^YFP/+^* mouse. YFP expressing *FoxJ1* lineage cells were stained by anti-GFP antibody to enhance fluorescence signal (green). S100β (red) labels GFP+ ependymal cells in the ventricular wall of the LV. (F) A coronal section of the SVZ of *Tie2-GFP* mouse. CD31 (red) labels GFP+ endothelial cells (green) in the SVZ. Scale bars: 20 µm (C,F), 10 µm (D,E). Nuclei were counterstained with Hoechst 33258 (blue).

The SVZ neurogenic niche contains several distinct cell types including NSCs, TAPs, astrocytes, and vascular endothelial cells (Figure 1A). In order to isolate each cell type in the SVZ niche, we took advantage of the genetic inducible fate mapping (GIFM) system and transgenic reporter mice that allow flow cytometry-based sorting of cells that express fluorescent proteins. First, we isolated the NSCs and TAPs based on their responsiveness to Shh signaling and *Gli1* expression in the SVZ [23]. Furthermore, the NSCs and TAPs could be separated based on the fact that only NSCs express GFAP [53]. Thus, NSCs, TAPs, and astrocytes from the SVZ can be separated by the combination of two labeling methods: *Gli1* lineage cells in the SVZ were labeled with tdTomato expression in *Gli1^CreER/+^;R26^tdTomato/+^* mice after tamoxifen treatment and GFAP+ cells were identified by GFP expression from the *hGFAP-GFP* mice [43]. In *Gli1^CreER/+^;hGFAP-GFP;R26^tdTomato/+^* mice, three weeks after tamoxifen treatment (Figure 1B), tdTomato expressing cells were mainly observed in the SVZ whereas GFP+ cells were observed in the SVZ immediately adjacent to the ependymal cell layer, as well as in the striatum (St) (Figure 1C). We examined the cellular identity of GFP+/tdTomato+ cells by using cell type-specific markers. Sox2 is expressed in dividing SVZ astrocytes (NSCs) and neural progenitors (TAPs) [54], [55]. Some GFP+/tdTomato+ cells in the SVZ were co-labeled with a NSC marker GFAP and Sox2 (Figure 1C and 1D, arrows). Based on the cellular location, transgenic marker expression, and immunohistochemical analysis, those are likely to be NSCs (type B cells) [53]. The GFP−/tdTomato+ cells in the SVZ were GFAP negative (Figure 1C, open arrowhead) and some of them expressed Sox2 (Figure 1D, open arrowhead) showing the characteristics of TAPs (type C cells). GFP+/tdTomato−/GFAP+ cells in the striatum (Figure 1C, arrowhead) are likely to be non-neurogenic astrocytes.

For the isolation of ependymal cells, we utilized *FoxJ1-Cre;R26^YFP/+^* mice that express YFP in ependymal cells and a small subset of astrocytes in the adult mouse SVZ [56]. In the *FoxJ1-Cre;R26^YFP/+^* mice, YFP+ *FoxJ1* lineage cells were located in the single outer cell layer of the lateral wall of the lateral ventricle and completely overlapped with a ependymal cell marker S100β expression [56] confirming their identity as ependymal cells (Figure 1E). YFP expression was also observed in the choroid plexus (not shown), which is consistent with the previous report that cells with the motile cilia express *FoxJ1* [45].

Finally, *Tie2-GFP* mice, in which the promoter of *Tie2,* a vascular endothelial-specific receptor tyrosine kinase, drives the *GFP* expression were used to isolate the vascular endothelial cells from the SVZ [16], [46]. GFP expression was observed in the vasculature of the entire brain including the SVZ and GFP expressing cells were labeled with a vascular endothelial cell marker CD31 (Figure 1F).

**Figure 2:**
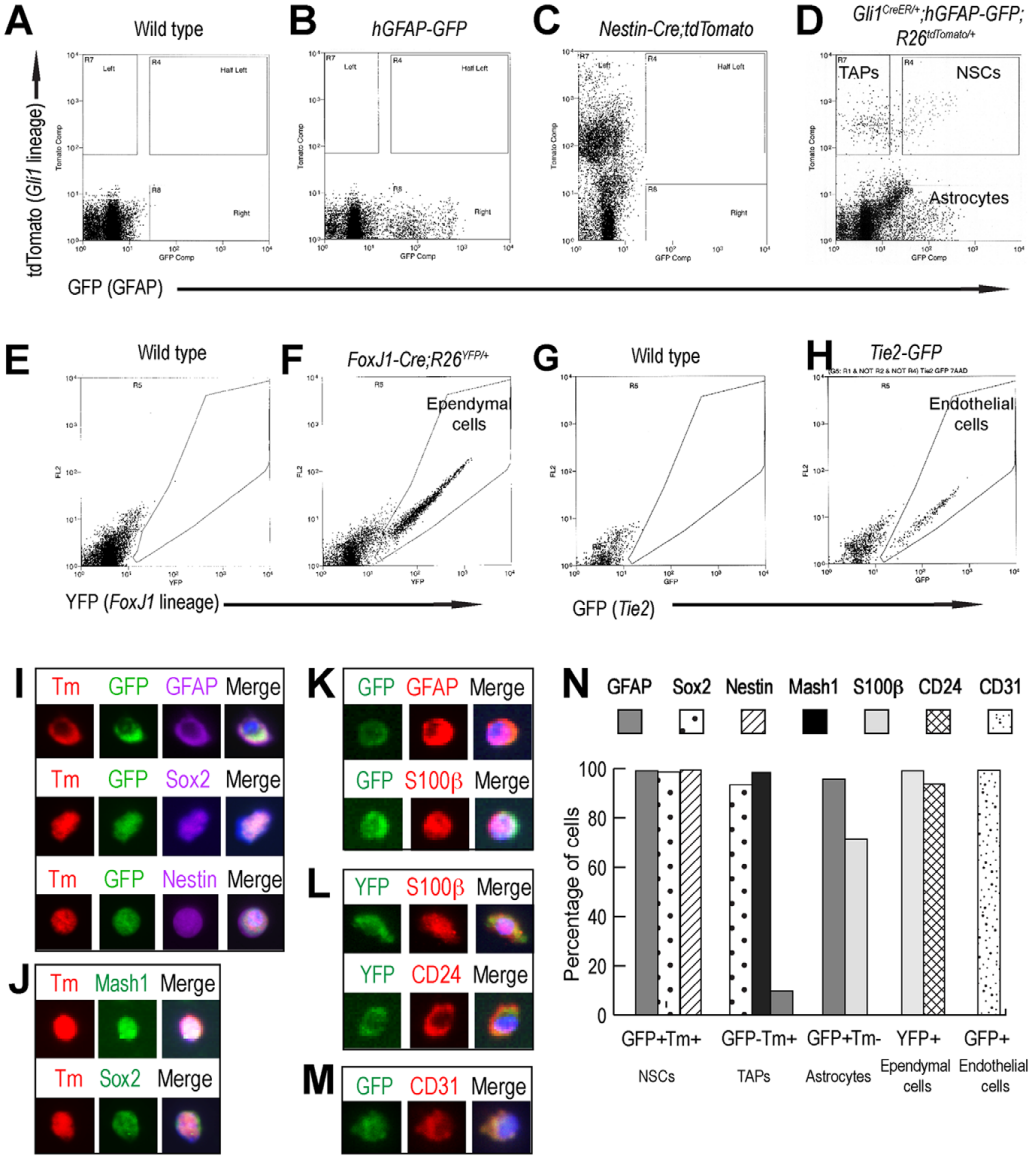
FACS isolation of SVZ niche cells. (A–D) FACS plots of dissociated SVZ cells from *Gli1^CreER/+^;hGFAP-GFP;R26^tdTomato/+^* mice. (A) Gate was set using wild-type mice as a negative control. (B) Gate setting for GFP using *hGFAP-GFP* mice. (C) Gate setting for tdTomato using *Nestin-Cre;R26^tdTomato/+^* mice. (D) FACS plot of the isolation of GFP+/tdTomato+ cells (NSCs), GFP−/tdTomato+ cells (TAPs), and GFP+/tdTomato− cells (astrocytes) from *Gli1^CreER/+^;hGFAP-GFP;R26^tdTomato/+^* mice. (E-F) FACS plots of dissociated SVZ cells from *FoxJ1-Cre;R26^YFP/+^* mice. (E) Gate setting for a negative control using wild-type mice. (F) FACS plot of the isolation of YFP+ ependymal cells. (G-H) FACS plots of dissociated SVZ cells from *Tie2-GFP* mice. (G) Gate setting for a negative control using wild-type mice. (H) FACS plot of the isolation of GFP+ endothelial cells. (I-N) Validation of FACS-Isolated NSC niche cells by immunofluorescent staining. Nuclei were stained with Hoechst 33258 (blue). (I) GFAP, Sox2, and Nestin label NSCs (GFP+/tdTomato+). (J) Mash1 and Sox2 label TAPs (GFP−/tdTomato+). (K) GFAP and S100β label astrocytes (GFP+/tdTomato−). (L) S100β and CD24 label ependymal cells (YFP+). (M) CD31 labels endothelial cells (GFP+). (N) Quantification of immunocytochemical validation. Data represent the ratio of total antibody marker expressing cells to transgenic marker expressing cells. The results are sum of at least 2 independent experiments.

As transgenic marker expressing cells showed the expected cell-type specific features *in vivo*, we next isolated NSCs and other putative niche cells by FACS, based on the expression of appropriate fluorescent reporter proteins as described above. First, the SVZ (the lateral wall of the lateral ventricle) of *Gli1^CreER/+^;hGFAP-GFP;R26^tdTomato/+^* mice were dissected, dissociated, and subjected to FACS three weeks after tamoxifen treatment (Figure 1B). From the SVZ of *Gli1^CreER/+^;hGFAP-GFP;R26^tdTomato/+^* mice, we were able to isolate Shh-responding NSCs based on their co-expression of GFP (GFAP+) and tdTomato (Gli1+) as well as TAPs (GFP−/tdTomato+) and non-neurogenic astrocytes (GFP+/tdTomato−) using FACS (Figure 2A–2D). For ependymal cells, YFP-expressing choroid plexus was first removed from *FoxJ1-Cre;R26^YFP/+^* mice before the dissection of the SVZ and YFP+ ependymal cells were isolated (Figure 2E and 2F). The SVZ of *Tie2-GFP* mice were also processed the same way for FACS and vascular endothelial cells were isolated based on GFP expression (Figure 2G and 2H). Since the choroid plexus secretes many soluble factors into the lumen of the lateral ventricle where the SVZ niche cells are in close contact, we also microdissected the choroid plexus of the lateral ventricle to identify potential factors that may influence NSC behaviors. Taken together, we were able to isolate each cell type in the SVZ neurogenic niche based on the cellular localization, immunohistochemical marker analysis, and transgenic markers.

### Validation of FACS-Isolated NSC Niche Cells

Next, we validated the identity of FACS-isolated SVZ niche cells. The sorted cells were attached on coated glass chamber slides immediately after FACS and processed for immunocytochemical analysis using cell type specific markers (Figure 2I–2N). GFAP is a specific marker of NSCs in the SVZ as well as astrocytes and is not expressed by TAPs [11]. Sox2 is expressed in dividing SVZ astrocytes (NSCs) and neural progenitors (TAPs) [54], [55]. Mash1 is predominantly expressed in TAPs and a subset of migrating neuroblasts [57]. S100β is a marker for ependymal cells as well as astrocytes [56]. CD24 is expressed in ependymal cells and also migrating immature neurons from the SVZ [58]. CD31 (PECAM-1) was used as a vascular endothelial cell marker [16]. Because each marker is not exclusively expressed by a single cell type, we used more than one marker for the cell-type analysis.

The majority of GFP+/tdTomato+ cells were positive for GFAP and Sox2 (Figure 2I and 2N). In addition, GFP+/tdTomato+ cells also showed Nestin expression [59] confirming their identity as NSCs (Figure 2I). Most GFP−/tdTomato+ cells expressed TAP markers Sox2 and Mash1 (93.3% and 98.3%, respectively), whereas only a subset of cells expressed GFAP (9.7%) (Figure 2J and 2N). Immunostaining of GFP+/tdTomato− cells showed that 95.6% of them were expressing S100β and 71.4% were expressing GFAP (Figure 2K and 2N), indicating that this population is highly enriched for astrocytes. The YFP+ cells isolated from the SVZ of *FoxJ1-Cre;R26^YFP/+^* mice expressed predominantly the ependymal cell markers S100β (99.0%) and CD24 (93.6%) (Figure 2L and 2N). Our immunohistochemical analysis indicates that 33.8% of isolated FoxJ1-YFP+ cells were positive for GFAP (data not shown), which is consistent with the published work that indicates a substantial portion of *FoxJ1* expressing cells were GFAP positive [56]. Our real time PCR analysis also detected the *Gfap* expression in FoxJ1-YFP+ cells was as high as in *Gli1*-tdTomato+/*GFAP*-GFP+ NSCs. However, ependymal cell-specific marker genes *S100β* and *Cd24* expression were 3.0 and 70.2-fold higher respectively in ependymal cells compared to NSCs (data not shown). These data indicate that the majority of *FoxJ1*-YFP+ cells exhibit ependymal cell characteristics. This result is mostly in agreement with a previous study, which showed that the relative expression of *Gfap* and *S100β* in isolated Prominin1+ ependymal cells are 0.4 and 3.0-fold respectively when compared to GFAP+/Prominin1+ NSCs [60]. Finally, the GFP+ cells isolated from the SVZ of *Tie2-GFP* mice expressed the vascular endothelial cell marker CD31 (99.1%) (Figure 2M and 2N). These results demonstrate that each niche cell type was indeed enriched by our FACS-based isolation protocol.

**Figure 3:**
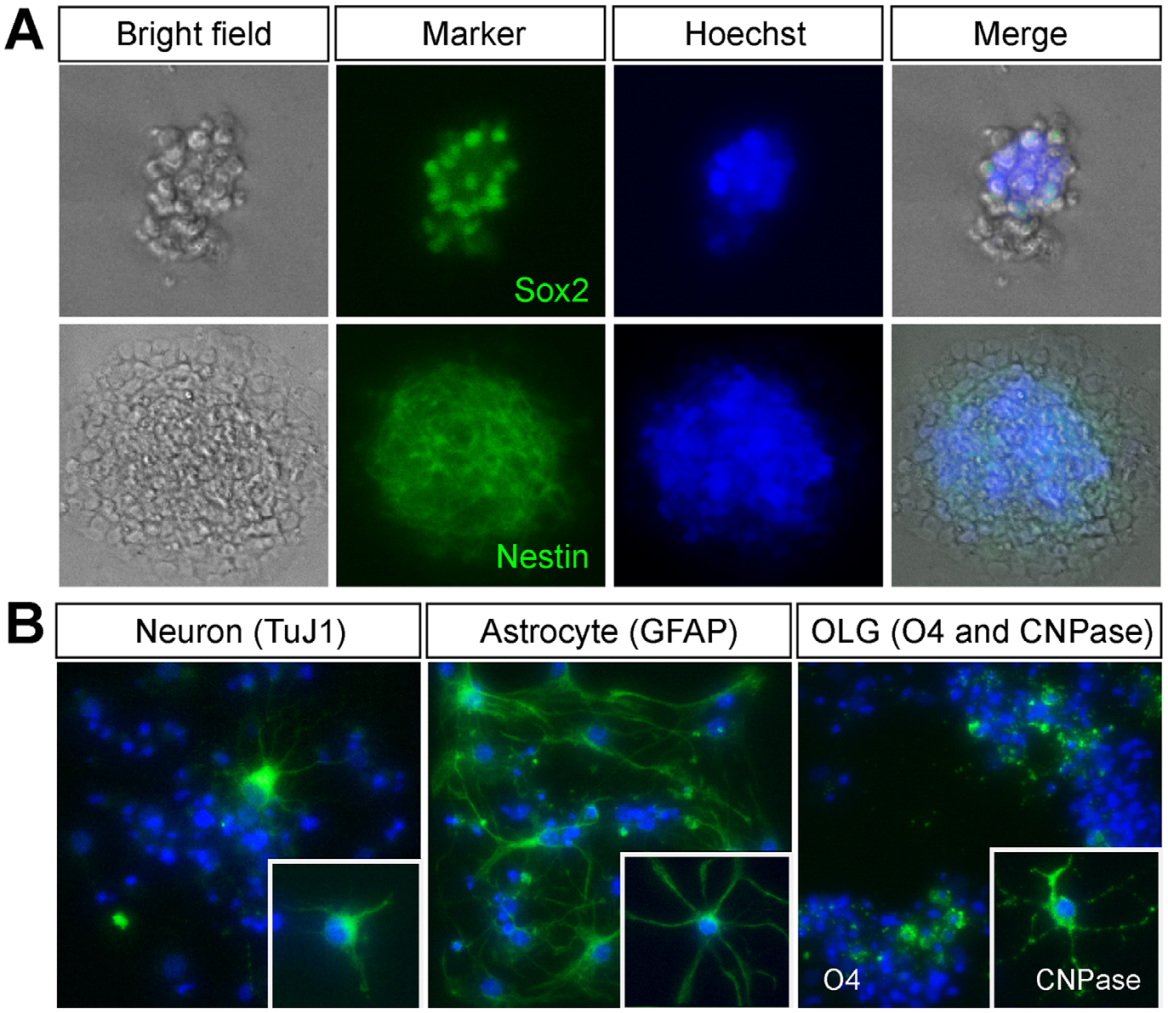
The self-renewal capacity and multipotency of GFP+/tdTomato+ cells. (A) FACS isolated GFP+/tdTomato+ cells from *Gli1^CreER/+^;hGFAP-GFP;R26^tdTomato/+^* mice formed neurospheres and express NSC markers Sox2 and Nestin. (B) Neurospheres or neurosphere-derived dissociated cells (insets) cultured in the differentiation medium differentiated into neurons (TuJ1+), astrocytes (GFAP+) and oligodendrocytes (O4+, inset: CNPase+). Immunofluorescent staining results were pseudocolored (green) and nuclei were stained with Hoechst 33258 (blue).

We further validated the NSC features, such as self-renewal capacity and multipotency, of isolated GFAP+/Gli1+ (GFP+/tdTomato+) cells by the neurosphere assay. In the presence of FGF2 and EGF in serum-free culture medium, GFP+/tdTomato+ cells formed neurospheres that expressed Sox2 and Nestin (Figure 3A). When 500 GFAP+/Gli1+ cells were directly sorted into a 96-well culture plate (3 wells) containing neurosphere culture medium, 34.7±5.7 (mean ± SD of 3 wells) neurospheres were observed 5 days later (6.9%). Due to the mosaic nature of GIFM, not all Gli1+ cells were labeled after tamoxifen treatment [23]. Thus GFAP+/Gli1− cells could contain Gli1-expressing (but not labeled) GFAP+ NSCs and produce neurospheres (data not shown). In addition, GFAP−/Gli1+ cells were capable of forming neurospheres since they are likely to be Shh-responding TAP cells [23], [61], [62]. When neurospheres derived from the GFAP+/Gli1+ (GFP+/tdTomato+) cells were induced to differentiate by adding serum and withdrawing FGF2 and EGF from the culture medium, cells changed their morphology and expressed markers for neurons (TuJ1), astrocytes (GFAP), and oligodendrocytes (O4 and CNPase) (Figure 3B). We observed that the NSC characteristics of GFP+/tdTomato+ cells were maintained up to the seventh passage, which was the highest passage number we tested in this study (data not shown). These results demonstrate that FACS-isolated GFP+/tdTomato+ cells were indeed the *in vivo* NSCs that retained the self-renewal capacity and could differentiate into multiple neural cell types *in vitro*.

### Secretory Molecule Expression Profile (SMEP) of NSC Niche Cells

To identify the secretory and cell-surface molecules expressed in each niche cell type, we employed the SST-REX screening method using the cDNA libraries obtained from each cell type of the SVZ niche. We initially identified a total of 307 cDNAs with the homology to known genes. Among them, we found the cDNAs of the secretory and cell-surface molecules as well as non-secretory molecules such as the nuclear proteins including cell cycle regulators (14.0%), cytoplasmic proteins (10.4%), mitochondrial proteins (7.2%), subcellular organelle membrane proteins (4.6%), and structural proteins (1.6%). We excluded those molecules from the profile and selected only the secretory and membrane-associated molecules by using the analysis tools, the Database for Annotation, Visualization and Integrated Discovery (DAVID) [63], NCBI Entrez gene (http://www.ncbi.nlm.nih.gov/gene), Mouse Genome Informatics (MGI, http://www.informatics.jax.org/genes.shtml), and Universal Protein Resource (UniProt, http://www.uniprot.org). Consequently, we obtained a list of 151 genes for the secretory molecule expression profile (SMEP) from 6 distinct NSC niche cell types (Table 1).

**Table 1:** Secretory molecule expression profiles (SMEP) of niche cells from the adult mouse SVZ.

Gene name	Gene symbol	Accession number
**NSC (Type-B cells)**		
sphingomyelin phosphodiesterase 2	*Smpd2*	NM_009213.2
sorbin and SH3 domain containing 1	*Sorbs1*	NM_001034962.1
apolipoprotein E	*Apoe*	NM_009696.3
2′,3′-cyclic nucleotide 3′ phosphodiesterase	*Cnp*	NM_001146318.1
cystatin C	*Cst3*	NM_009976.3
neural cell adhesion molecule 1	*Ncam1*	NM_001081445.1
leucine zipper protein 2	*Luzp2*	NM_178705.5
ATPase, Na+/K+ transporting, alpha 2 polypeptide	*Atp1a2*	NM_178405.3
solute carrier family 24 member 6	*Slc24a6*	NM_001177594.1
milk fat globule-EGF factor 8 protein	*Mfge8*	NM_008594.2
ribosomal protein SA (Rpsa), similar to 67 kda laminin receptor	*Rpsa*	NM_011029.4
transmembrane emp24 protein transport domain containing 9	*Tmed9*	NM_026211.3
receptor-type tyrosine-protein phosphatase zeta	*Ptprz1*	NM_001081306.1
carbonic anhydrase	*Car11*	NM_009800.4
scrapie responsive gene 1	*Scrg1*	NM_009136.3
HtrA serine peptidase 1	*Htra1*	NM_019564.3
NECAP endocytosis associated 2	*Necap2*	NM_025383.3
mesoderm development candidate 2	*Mesdc2*	NM_023403.3
prosaposin	*Psap*	NM_001146124.1
**transit-amplifying cells (type-C cells)**		
SPARC-like 1	*Sparcl1*	NM_010097.4
interleukin 1 receptor, type II	*Il1r2*	NM_010555.4
cytotoxic T lymphocyte-associated protein 2 alpha	*Ctla2a*	NM_007796.2
epidermal growth factor-containing fibulin-like extracellular matrix protein 1	*Efemp1*	NM_146015.2
G protein-coupled receptor 116	*Gpr116*	NM_001081178.1
transmembrane protein 9	*Tmem9*	NM_001160145.1
limbic system-associated membrane protein	*Lsamp*	NM_175548.3
CD63 antigen	*Cd63*	NM_007653.3
CD81 antigen	*Cd81*	NM_133655.2
transthyretin	*Ttr*	NM_013697.5
ATP-binding cassette, sub-family A (ABC1), member 4	*Abca4*	NM_007378.1
apolipoprotein E	*Apoe*	NM_009696.3
neurotrimin	*Hnt*	NM_172290.3
lysosomal-associated membrane protein 2	*Lamp2*	NM_010685.3
transmembrane protein 4	*Tmem4*	NM_019953.1
matrix-remodelling associated 8	*Mxra8*	NM_024263.4
folate receptor 1 (adult)	*Folr1*	NM_008034.2
serine protease inhibitor, Kunitz type 2	*Spint2*	NM_001082548.1
protocadherin 17	*Pcdh17*	NM_001013753.2
clathrin, light polypeptide (Lca)	*Clta*	NM_001080384.1
inhibin beta-A	*Inhba*	NM_008380.1
cystatin C	*Cst3*	NM_009976.3
cortexin 3	*Ctxn3*	NM_001134697.1
apolipoprotein D	*Apod*	NM_007470.3
solute carrier family 1 member 1	*Slc1a1*	NM_009199.2
glutamate receptor ionotropic AMPA4	*Gria4*	NM_001113181.1
neurotrophic tyrosine kinase receptor type 2	*Ntrk2*	NM_008745.2
cathepsin D	*Ctsd*	NM_009983.2
ring finger protein 128	*Rnf128*	NM_023270.5
signal-regulatory protein alpha	*Sirpa*	NM_001177646.1
fibroblast growth factor receptor-like 1	*Fgfrl1*	NM_054071.2
transmembrane emp24 protein transport domain containing 5	*Tmed5*	NM_028876.2
**Astrocyte**		
protein disulfide isomerase associated 3	*Pdia3*	NM_007952.2
DnaJ (Hsp40) homolog, subfamily C, member 3A	*Dnajc3a*	NM_008929.3
Fc receptor, IgG, alpha chain transporter	*Fcgrt*	NM_010189.3
lysosomal-associated membrane protein 1	*Lamp1*	NM_010684.2
SPARC-like 1	*Sparcl1*	NM_010097.4
CD81 antigen	*Cd81*	NM_133655.2
receptor (calcitonin) activity modifying protein 2	*Ramp2*	NM_019444.2
apolipoprotein E	*Apoe*	NM_009696.3
calsequestrin 2	*Casq2*	NM_009814.2
interferon (alpha and beta) receptor 2	*Ifnar2*	NM_001110498.1
glutamate receptor, ionotropic, AMPA4 (alpha 4)	*Gria4*	NM_001113181.1
phosphoinositide-3-kinase interacting protein 1	*Pik3ip1*	NM_178149.4
apoptosis-related protein 3 isoform 1	*Apr3*	NM_027855.3
secretogranin III	*Scg3*	NM_009130.3
phosphatidic acid phosphatase type 2 domain containing 2	*Ppapdc2*	NM_028922.3
cystatin C	*Cst3*	NM_009976.3
arginine-rich, mutated in early stage tumors	*Armet*	NM_029103.3
tetraspanin 17	*Tspan17*	NM_028841.3
syndecan	*Sdc2*	NM_008304.2
secreted acidic cysteine rich glycoprotein	*Sparc*	NM_009242.4
transthyretin	*Ttr*	NM_013697.5
limbic system-associated membrane protein	*Lsamp*	NM_175548.3
leucine zipper protein 2	*Luzp2*	NM_178705.5
tissue inhibitor of metalloproteinase 4	*Timp4*	NM_080639.3
solute carrier family 1 (glial high affinity glutamate transporter) member 3	*Slc1a3*	NM_148938.3
neuron-glia-CAM-related cell adhesion molecule	*Nrcam*	NM_176930.4
**Ependymal cells**		
apolipoprotein E	*Apoe*	NM_009696.3
cadherin-related family member 3	*Cdhr3*	NM_001024478.1
adenylosuccinate synthetase like 1	*Adssl1*	NM_007421.2
vitronectin	*Vtn*	NM_011707.2
solute carrier family 39 member 6	*Slc39a6*	NM_139143.3
stromal interaction molecule 2	*Stim2*	NM_001081103.2
layilin	*Layn*	NM_001033534.1
transthyretin	*Ttr*	NM_013697.5
folate receptor 1 adult)	*Folr1*	NM_008034.2
cystatin C	*Cst3*	NM_009976.3
ras-related protein Rab-31	*Rab31*	NM_133685.2
transmembrane protein 9	*Tmem9*	NM_001160145.1
solute carrier family 29 (nucleoside transporters) member 4	*Slc29a4*	NM_146257.2
RAB11B, member RAS oncogene family	*Rab11b*	NM_008997.3
retinoic acid receptor responder (tazarotene induced) 2	*Rarres2*	NM_027852.2
**choroid plexus**		
transthyretin	*Ttr*	NM_013697.5
solute carrier family 16 (monocarboxylic acid transporter) member 8	*Slc16a8*	NM_020516.2
clusterin	*Clu*	NM_013492.2
apolipoprotein E	*Apoe*	NM_009696.3
lysosomal-associated membrane protein 1	*Lamp1*	NM_010684.2
CD81 antigen	*Cd81*	NM_133655.2
serine protease inhibitor, Kunitz type 2	*Spint2*	NM_001082548.1
lysosomal-associated membrane protein 2	*Lamp2*	NM_010685.3
secreted phosphoprotein 1	*Spp1*	NM_009263.3
ADAMTS-like protein 1	*Adamtsl1*	NM_029967.3
folate receptor 1 (adult)	*Folr1*	NM_008034.2
CD82 antigen	*Cd82*	NM_007656.4
apolipoprotein D	*Apod*	NM_007470.3
Kazal-type serine peptidase inhibitor domain 1	*Kazald1*	NM_178929.4
heat shock protein 90, beta (Grp94) member 1	*Hsp90b1*	NM_011631.1
solute carrier family 12, member 2	*Slc12a2*	NM_009194.3
prostaglandin D2 synthase (brain)	*Ptgds*	NM_008963.2
5-hydroxytryptamine (serotonin) receptor 2C	*Htr2c*	NM_008312.4
prolactin receptor	*Prlr*	NM_011169.5
platelet derived growth factor receptor, alpha polypeptide	*Pdgfra*	NM_011058.2
ectonucleotide pyrophsphatase/phosphodiesterase 2	*Enpp2*	NM_015744.2
insulin-like growth factor binding protein 2	*Igfbp2*	NM_008342.2
praja2, RING-H2 motif containing	*Pja2*	NM_144859.2
tissue inhibitor of metalloproteinase 3	*Timp3*	NM_011595.2
protein disulfide isomerase associated 6	*Pdia6*	NM_027959.3
cathepsin B	*Ctsb*	NM_007798.2
heat shock protein 5	*Hspa5*	NM_001163434.1
coiled-coil domain containing 56	*Ccdc56*	NM_026618.2
vitronectin	*Vtn*	NM_011707.2
cathepsin d	*Ctsd*	NM_009983.2
cysteine-rich with EGF-like domains 2	*Creld2*	NM_029720.2
integral membrane protein 2B	*Itm2b*	NM_008410.2
**Endothelial cells**		
talin 1	*Tln1*	NM_011602.5
cadherin 5	*Cdh5*	NM_009868.4
amyloid beta A4 protein	*App*	NM_007471.3
angiopoietin-related protein 2	*Angptl2*	NM_011923.4
retinoic acid early-inducible protein 1-alpha	*Raet1a*	NM_009016.1
angiopoietin 2	*Angpt2*	NM_007426.3
amyloid beta (A4) precursor-like protein 2	*Aplp2*	NM_009691.2
CD34 antigen	*Cd34*	NM_133654.3
glycine receptor subunit alpha 1	*Glra1*	NM_020492.3
guanine nucleotide-binding protein subunit beta-2-like 1	*Gnb2l1*	NM_008143.3
insulin-like growth factor 1 receptor	*Igf1r*	NM_010513.2
integrin beta 4	*Itgb4*	NM_133663.2
laminin subunit beta 1	*Lamb1*	NM_008482.2
plexin D1	*Plxnd1*	NM_026376.3
prolow-density lipoprotein receptor-related protein 1	*Lrp1*	NM_008512.2
solute carrier family 22 member 18	*Slc22a18*	NM_008767.2
SPARC-related modular calcium-binding protein 2	*Smoc2*	NM_022315.2
sphingosine 1-phosphate receptor 4	*S1pr4*	NM_010102.2
tyrosine-protein kinase receptor	*Tie1*	NM_011587.2
vascular cell adhesion molecule 1	*Vcam1*	NM_011693.3
cell adhesion molecule 1	*Cadm1*	NM_018770.3
cell adhesion molecule 2	*Cadm2*	NM_178721.4
cell adhesion molecule 4	*Cadm4*	NM_153112.3
endothelial-specific receptor tyrosine kinase	*Tek*	NM_013690.2
endomucin	*Emcn*	NM_016885.2
endothelial protein C receptor	*Procr*	NM_011171.2
selectin, endothelial cell	*Sele*	NM_011345.2
FXYD domain-containing ion transport regulator 5	*Fxyd5*	NM_008761.3
integrin alpha 1	*Itga1*	NM_001033228.3
integrin alpha 2b	*Itga2b*	NM_010575.2
integrin beta 1	*Itgb1*	NM_010578.2
laminin subunit alpha 3	*Lama3*	NM_010680.1
laminin subunit alpha 4	*Lama4*	NM_010681.4
low-density lipoprotein receptor-related protein 5	*Lrp5*	NM_008513.3
lysosome-associated membrane glycoprotein 2	*Lamp2*	NM_010685.3
matrix metalloproteinase 14	*Mmp14*	NM_008608.3
neuromedin-U receptor 2	*Nmur2*	NM_153079.4
platelet derived growth factor receptor beta	*Pdgfrb*	NM_008809.2
plexin A1	*Plxna1*	NM_008881.2
proheparin-binding EGF-like growth factor	*Hbegf*	NM_010415.2
receptor-type tyrosine-protein phosphatase C	*Ptprc*	NM_011210.3
sodium- and chloride-dependent glycine transporter 2	*Slc6a5*	NM_148931.3
Y+L amino acid transporter 1	*Slc7a7*	NM_011405.3
solute carrier family 8 member 2	*Slc8a2*	NM_148946.2
secreted acidic cysteine rich glycoprotein	*Sparc*	NM_009242.4
syndecan 4	*Sdc4*	NM_011521.2
tenascin XB	*Tnxb*	NM_031176.2
transmembrane protein 59	*Tmem59*	NM_029565.3
tumor necrosis factor ligand superfamily member 9	*Tnfsf9*	NM_009404.3
tumor necrosis factor ligand superfamily member 14	*Tnfsf14*	NM_019418.2
tumor necrosis factor receptor superfamily member 18	*Tnfrsf18*	NM_009400.2
protocadherin 12	*Pcdh12*	NM_017378.2
dolichyl-phosphate (UDP-N-acetylglucosamine) acetylglucosaminephosphotransferase 1 (GlcNAc-1-P transferase)	*Dpagt1*	NM_007875.2
von Willebrand factor	*Vwf*	NM_011708.3

**Figure 4:**
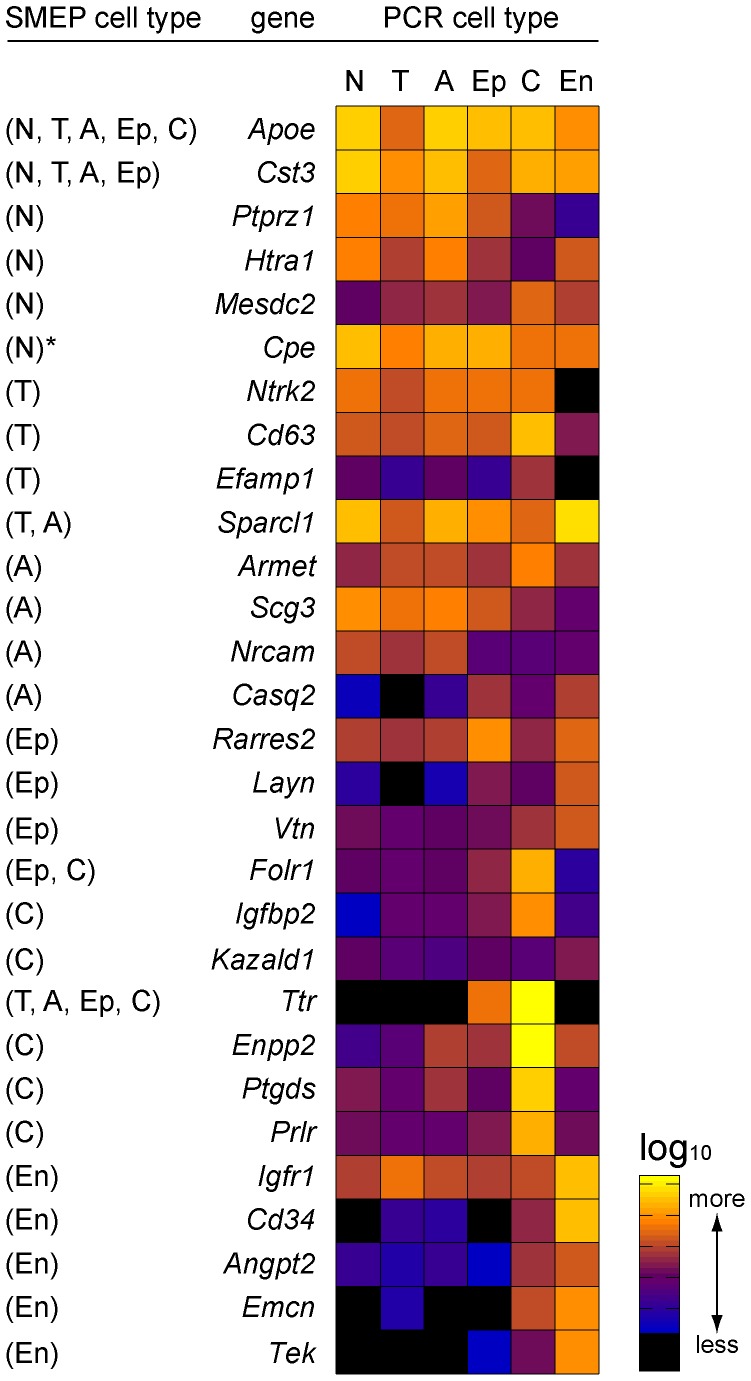
Confirmation of genes in SMEP by qRT-PCR. A heat map represents the expression levels for 29 selected genes using qRT-PCR from 151 SST-REX identified genes and 81 potential NSC SMEP by microarray. Each colored grid in the heat map represents the relative abundance of the transcript compared to the *Gapdh* level. Each gene was identified by either single or multiple niche cell type SMEP (parentheses on the left column). N: NSCs, T: TAPs, A: astrocytes, Ep: ependymal cells, C: choroid plexus, En: endothelial cells. * indicates the SMEP from microarray experiment.

To assess the cell type-specificity of our SMEP, we analyzed the relative expression level of 29 genes in each niche cell type by qRT-PCR. The threshold cycle (Ct) value of each gene was normalized by the Ct value of *Gapdh* and the normalized values were plotted in a heat map for each cell type (Figure 4). SMEP showed that some genes were expressed in multiple niche cell types, for example *Apoe* (NSCs, TAPs, astrocytes, ependymal cells, and choroid plexus) and *Cst3* (NSCs, TAPs, astrocytes, and ependymal cells). Our qRT-PCR results demonstrated that *Apoe* and *Cst3* were highly expressed in all 6 niche cell types. The heat map showed that the genes identified by the choroid plexus or endothelial cell SMEP were highly expressed by the respective cell type. For example, *Fol1, Igfbp2, Ttr, Enpp2, Ptgds*, and *Prlr* were screened by SST-REX from the choroid plexus and were specifically expressed in the choroid plexus. The genes identified by other cell type SMEP (NSCs, TAPs, astrocytes, and ependymal cells) were expressed in the respective cell type as well as other cell types. Overall, this shows that the genes in each cell type SMEP are indeed expressed by the respective SVZ niche cell type.

In order to better understand whether these identified genes are potentially involved in neurogenic niche signaling, we performed gene functional annotation analysis using DAVID (Table 2). The functional categorization showed that 32 genes (21.2%) are involved in cell adhesion including the extracellular matrix (ECM)/receptor interaction, cell-cell adhesion, and the regulation of cell migration. Genes that encode proteins related to growth factor binding, carbohydrate binding, and receptor-based signaling pathway were also identified. 16.6% of the identified genes are known to be responsible for membrane transport of ions, hormones, and metabolic materials. Together, these analyses indicate that many of the identified genes in our SMEP are indeed known to have roles in cell-cell, ECM-cell, and ligand-receptor interactions.

**Table 2:** Top enriched annotation terms for secretory molecule expression profile (SMEP) from subventricular zone niche cells.

Data source	Annotation term	%	p value
GOTERM_BP_FAT	Cell adhesion	21.2	2.7E−17
GOTERM_MF_FAT	Growth factor binding	5.3	2.2E−6
GOTERM_MF_FAT	Carbohydrate binding	7.9	6.8E−5
GOTERM_BP_FAT	Transmembrane receptor protein tyrosine kinasesignaling pathway	5.3	1.3E−3
SP_PIR_KEYWORDS	Transport	16.6	3.0E−3

Since we did not have an appropriate mouse line to isolate neuroblasts as comparable to the cell type-specific mouse lines used in this study for other niche cells, we decided to focus our gene expression profiling using NSCs, TAPs, astrocytes, ependymal cells, and vascular endothelial cells as the SVZ niche cells. Pennartz et al. used PSA-NCAM+ cells for the serial analysis of gene expression (SAGE) to identify genes enriched in the neuroblasts [64]. Therefore, we have extracted the information embedded in this study to expand our repertoire of signaling molecules in the SVZ neurogenic niche (Table S3).

### Potential SMEP of NSC by Microarray Method

The SST-REX screening method has a caveat that there are secreted proteins without a signal sequence such as fibroblast growth factors (FGFs). Moreover, the profile we obtained in this study may not represent the complete list of all potential secretory and cell-surface molecules in the niche. Thus, we combined the SST-REX with another high throughput screening approach, the microarray expression analysis, to extend the SMEP results. While the Shh-responding and GFAP expressing (GFAP+/Gli1+) cells in the adult SVZ are NSCs [23], GFAP+/Gli1+ cells in the adult cerebellum are non-neurogenic Bergmann glia [65], [66]. Thus, we reasoned that the molecules specifically involved in regulating NSC behaviors would be differentially expressed by GFAP+/Gli1+ cells in the SVZ compared to GFAP+/Gli1+ cells in the cerebellum. We isolated GFAP+/Gli1+ cells in the SVZ and in the cerebellum, separately, from *Gli1^CreER/+^;hGFAP-GFP;R26^tdTomato/+^* mice and performed Affymetrix microarray analysis. Gene expression profiling showed that 382 genes were differentially expressed between SVZ and cerebellum (p<0.01 with at least 4.0-fold change). 106 genes were upregulated and 276 genes were downregulated in the SVZ GFAP+/Gli1+ cells compared to cerebellar GFAP+/Gli1+ cells (data not shown). From these data, we identified 84 genes encoding secretory or transmembrane proteins (30 up and 54 down in the SVZ, Table S4), which provided us an additional set of secretory and cell-surface molecules that are uniquely expressed in the Shh-responding NSCs. We have not only identified differentially expressed genes, but also determined the most highly expressed genes in GFAP+/Gli1+ SVZ cells. We selected the top 2.5% probesets based on whole array intensities distribution, and then filtered with one-sided Wilcoxon’s Signed Rank test p-value to confirm the transcripts expressed on the array. From a total of 740 highly expressed genes in GFAP+/Gli1+ SVZ cells (1008 transcripts), we identified 127 genes encoding secretory or transmembrane proteins (Table S5).

### Functional Validation of Identified Molecules *in vitro*

Next, we selected a few candidate molecules and examined their potential function as NSC niche signals. We used the neurosphere model system to assess the proliferation and differentiation potential of NSCs in response to these soluble factors *in vitro.* The neurosphere model has been widely used to test the effect of soluble factors on NSC behaviors *in vitro* [17], [25], [26], [34]. We quantified the neurosphere number and the size (diameter) to determine the effect of the candidate molecules on the neurosphere formation capacity and the mitogenic potency, respectively. We also estimated total number of cells by XTT assay, which is a tetrazolium-based colorimetric assay that evaluates the viability and the proliferation of cells [67].

**Figure 5:**
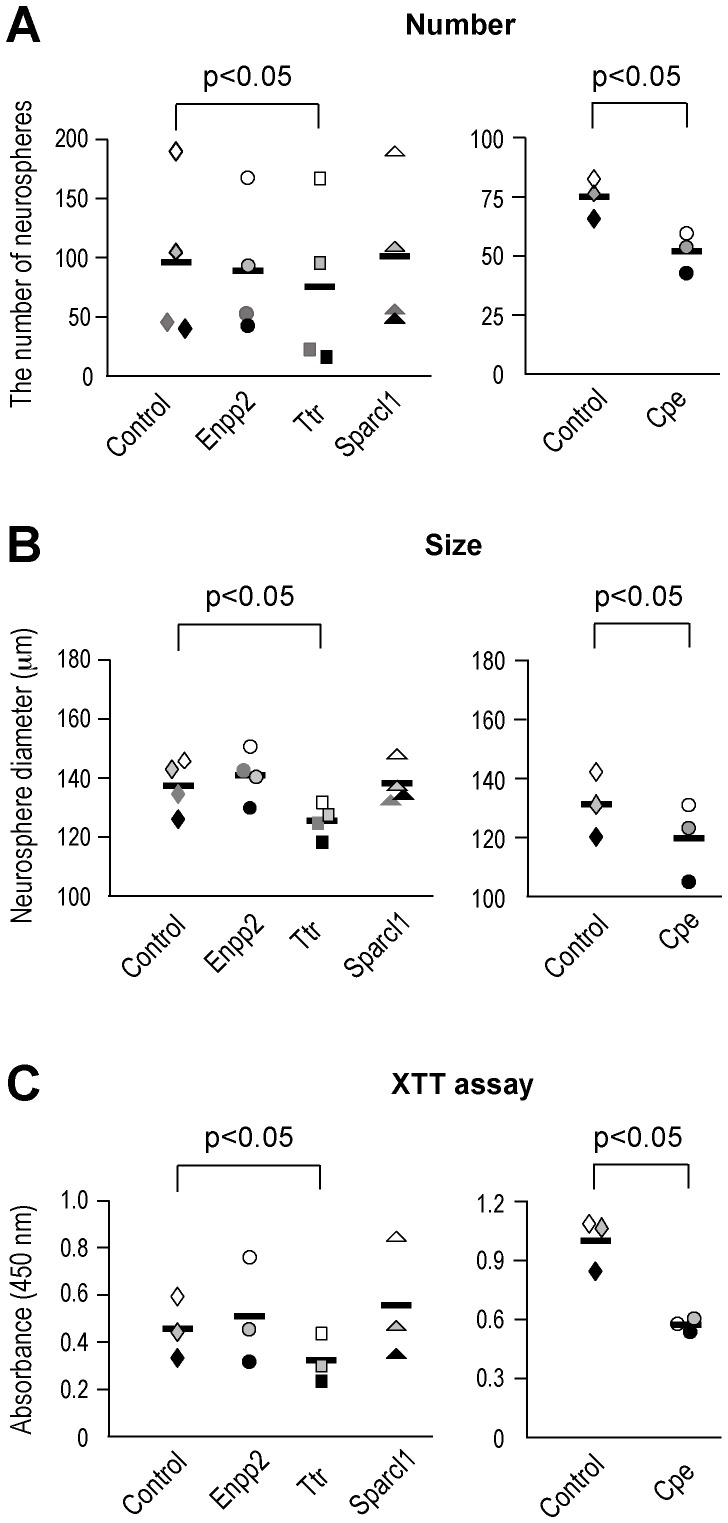
Functional validation of identified molecules *in vitro* neurosphere culture. (A) The number of neurospheres. In the presence of Ttr and CPE, fewer neurospheres formed, while Enpp2 and Sparcl1 treatment did not show significant differences (Ttr: p = 0.012, n = 4; CPE: p<0.0001, n = 3). (B) The size of neurospheres determined by the neurosphere diameter. In the presence of Ttr and CPE, smaller neurospheres were formed, while Enpp2 and Sparcl1 treatment did not show significant differences (Ttr: p = 0.0072, n = 4; CPE: p = 0.031, n = 3). (C) XTT cell proliferation assay. Ttr and CPE treatment resulted in less number of total cells in culture wells compared to the control, while Enpp2 and Sparcl1 treatment did not show significant differences (Ttr: p = 0.017, CPE: p = 0.020; n = 3). Each symbol represents the mean of triplicates from each independent experiment. Bars are the mean of 3 or 4 such independent experiments. Symbols with the same color in the graph are the data set of each independent experiment.

Among the identified genes, 4 candidates, *Enpp2, Ttr, Sparcl1*, and *CPE*, were selected based on their experimental suitability, i.e., solubility in the cell culture medium, and a previously reported role in cell proliferation and differentiation. First, Enpp2 (ectonucleotide pyrophosphatase/phosphodiesterase 2, also known as autotaxin) from the choroid plexus SMEP is a secreted enzyme [68], and converts lysophosphatidylcholine (LPC) into the lipid signaling molecule lysophophatidic acid (LPA) [69]. LPA in turn interacts with its G protein-coupled receptors to exert various signaling functions such as cell proliferation, survival, migration, and production of second messengers in many cells [70] including neural progenitor cells [71]. However, the addition of recombinant Enpp2 in the culture medium did not affect the neurosphere number, size, or total cell number in our study (Figure 5). Since the lack of Enpp2 effect could result from the insufficient amount of substrate LPC in the medium, we included LPC in the neurosphere culture medium. The combinatorial treatment of Enpp2 and LPC resulted in increased number and size of neurospheres compared to Enpp2 alone, but the difference was not statistically significant compared to the control (p = 0.080 for the number and p = 0.18 for the size of neurospheres, data not shown). It has been shown that while LPA treatment could generate a comparable number of neurospheres as EGF+FGF2 treatment alone, no significant additive effect was observed when LPA was combined with EGF+FGF2 treatment [72]. Therefore, the lack of Enpp2-induced proliferation could also be due to the fact that our culture medium was already saturated for the mitotic signals provided by EGF and FGF2.

Next, we investigated the effects of transthyretin (Ttr), which was identified from the TAPs, astrocytes, ependymal cells and choroid plexus SMEP. Ttr is the major binding protein for the thyroxine (T_4_), a form of thyroid hormones, in the CSF and is responsible for thyroid hormone supply to the brain [73]. In the brain, T_4_ is converted to a bioactive form triiodothyronine (T_3_) by a brain-specific deiodinase. Thyroid hormones play very important roles in brain development [74] and in adult neurogenesis as the experimental hypothyroidism resulted in reduced NSC proliferation and neuroblast migration with increased apoptosis [75], [76]. After the treatment of the exogenous Ttr, fewer neurospheres were formed (p = 0.012) and the average neurosphere diameter was significantly smaller than the control (p = 0.0072). Total cell number was also reduced following Ttr treatment, which was estimated by XTT assay (p = 0.017) (Figure 5). Thus, it appears that Ttr negatively affects neurosphere formation and cell proliferation in our neurosphere culture model, possibly by sequestering the bioactive form of thyroid hormone in the medium.

In addition, we tested the function of SPARC-like 1 (Sparcl1, also known as Hevin), which was identified from the TAPs and astrocytes SMEP. Sparcl1 is a secreted extracellular matrix (ECM) glycoprotein known to be involved in cell adhesion, migration, and ECM remodeling after brain injuries [77]–[79]. Sparcl1 expression is often found down-regulated in various cancer cells and it may act as a negative regulator of cell proliferation [80]. Sparcl1 is highly expressed in the brain [81], primarily in endothelial cells and astrocyte lineage cells, including radial glia in the developing cortex and Bergmann glia in the cerebellum. When we added recombinant Sparcl1 in the culture medium, we did not observe any effect on the size nor the number of neurospheres. Because the main function of Sparcl1 is related to ECM-based cell migration and remodeling, it might not have a significant effect on neurospheres, which are in suspension in culture media. The fact that Sparcl1 was identified in endothelial cells and astrocytes [79], which form a migratory scaffold in the rostral migratory stream [82], raises the possibility that it may be involved in the migration of neuroblasts.

Our microarray result showed that carboxypeptidase E (CPE) was highly expressed in the SVZ compared to the cerebellum. CPE was originally reported as a soluble peptidase that hydrolyzes C-terminal basic amino acids of peptides cleaved from pro-neuropeptides and pro-peptide hormones to produce the bioactive peptides in secretory vesicles [83]. Besides the peptidase activity, other various non-enzymatic functions of CPE have been reported [84]. Whereas the soluble form of CPE acts as a processing enzyme, the membrane associated form of CPE acts as a sorting receptor at the trans-Golgi network to target prohormones and proBDNF to the regulated secretory pathway [85], [86]. Additionally, wild-type CPE has been shown to promote proliferation and has an anti-metastatic effect in glioma cells [87]. However, an N-terminal truncated isoform of CPE (CPE-ΔN) was found to drive tumor progression in different types of cancer [88]. In the nervous system, CPE exerts its function in neuroprotection [89] and dendrite development [90], [91]. Our experiment showed that in the presence of recombinant CPE, fewer neurospheres were formed (p<0.0001) and the average neurosphere diameter was significantly smaller than the control (p = 0.031). XTT cell proliferation assay further confirmed that the total number of cells was also reduced following CPE treatment (p = 0.020) (Figure 5). Overall, only Ttr or CPE, but not Enpp2 or Sparcl1, exhibited their negative effects on the cell proliferation and formation of neurospheres.

Next, we tested whether these molecules could affect the differentiation potential of the neurospheres. After adding Enpp2, Ttr, Sparcl1, or CPE in the differentiating medium for 5 days, we assessed the markers for differentiated neural cell types (*TuJ1*: neurons, *Gfap*: astrocytes, *Olig2*: oligodendrocytes) in each treated culture condition by qRT-PCR and found no significant differences compared to the control culture (data not shown). These results suggest that Enpp2, Ttr, Sparcl1, and CPE are not involved in the differentiation capacity of the neurospheres derived from the adult SVZ.

## Discussion

Neural stem cells (NSCs) are the principal source of all cells found in the nervous system. Embryonic NSCs have the potential to generate the full array of neurons and glia in the brain. In contrast, adult NSCs are restricted in their potential and produce neurons in only specific regions of the forebrain. However, even adult NSCs can be coaxed to produce a broad range of neural cells in the appropriate culture conditions. In the postnatal mammalian brain, NSCs are retained in the specialized microenvironment called the neurogenic niche. The various environmental cues provided by the niche converge onto NSCs, which in turn utilize their intrinsic factors to respond appropriately for proper control of neurogenesis. In this study, we investigated the nature of the communication between NSCs and their environment that may control stem cell maintenance, differentiation, and lineage specification. We used the signal sequence trap method for the systematic exploration of autocrine/paracrine as well as cell-cell contact-based signaling factors derived not only from NSCs but also from their neighboring niche cells. Here, we present the SMEPs of NSCs and other niche cell types, including TAPs, non-neurogenic astrocytes, ependymal cells, vascular endothelial cells, and the choroid plexus.

Because multiple niche cell types are tightly associated in the SVZ, the isolation of each SVZ niche cell type at high purity is the critical first step for obtaining the cell type-specific expression profiles. There have been numerous attempts to isolate NSCs and surrounding niche cells in highly purified forms by FACS. Mostly they relied on the use of a single transgenic reporter mouse line [92], [93] and/or labeling cell surface markers or intracellular markers through permeabilization [94], [95]. Since NSCs and niche cells do not have a single bona fide marker, another study used a dual-labeling method to enrich the NSC population [62]. These studies demonstrate the value of the transgenic mice for isolation of specific SVZ cell types, especially in combination with multiple overlapping markers. However, the additional steps required for labeling with ligands or antibodies result in a prolonged incubation period of freshly dissociated cells, raising the possibility of altering the dynamics of gene expression profiles in processed cells. Our approach combined the genetic inducible fate mapping system and transgenic reporter mice (*Gli1^CreER/+^;hGFAP-GFP;R26^tdTomato/+^*, *FoxJ1-Cre;R26^YFP/+^*, and *Tie2-GFP* mice) to isolate NSCs, TAPs, astrocytes, ependymal cells and endothelial cell from the SVZ. It is a highly efficient and reproducible method to isolate specific cell types because it is based on the genetic labeling and nascent fluorescent protein expression and does not require additional processing steps. Using this method, we were able to isolate highly enriched cell types from the SVZ niche as our validation experiments demonstrate.

Previous studies demonstrated that a subset of FoxJ1+ cells in the SVZ express GFAP and these FoxJ1+/GFAP+ cells have NSC-like features such as neurosphere formation [56]. One scenario is that a subset of dividing SVZ astrocytes (GFAP+) incorporates within the ependymal cell layer and those incorporated cells acquire antigenic and morphological properties of mature ependymal cells, which is more often observed in the aged brain [96]. Interestingly, during this transition state, incorporated cells co-express GFAP as well as ependymal cell markers [97]. Thus, it is likely that a subset of newly born SVZ astrocytes incorporate into the ependymal cell layer, gradually lose GFAP expression, while obtaining ependymal cell features [96]. Together, our results indicate that the *FoxJ1*-YFP+ cell population contains GFAP-expressing cells but the majority of *FoxJ1*-YFP+ cells are ependymal cells expressing CD24 and S100β.

Our SMEP data include well-known or previously identified cell type-specific genes such as *Ptgds* (choroid plexus) [98] and *Cd34* (endothelial cells) [99]. Besides these two genes, the expression patterns of multiple genes we identified by SST-REX have been reported in certain SVZ cell types (Table S3). Additionally, we identified some novel genes that are enriched in each cell type that could have potential relevance for the niche signals. For example, we showed that the exogenous treatment of Ttr on neurosphere culture resulted in less neurosphere formation and reduced cell proliferation. Although Ttr is the major thyroid hormone (T_4_) supplier in the brain, there was only T_3_ in our neurosphere culture medium (B-27 supplement, Invitrogen). Because Ttr can also bind to T_3_ but with a lower affinity than T_4_ [100], Ttr might bind to and sequester T_3_, thereby reducing bioavailability of T_3_ in the culture medium. In agreement with our result, it was shown that an antithyroid drug that induced experimental hypothyroidism reduces the NSC proliferation [76]. Interestingly, T_4_ and T_3_ levels in the brain parenchyma of Ttr null mice were not reduced [101] and the number of dividing cells in the SVZ of adult Ttr null mice is comparable to that of Ttr wild-type mice [102], suggesting the possibility of a thyroid hormone-independent role for Ttr. Because the function of Ttr on NSC behaviors is largely unknown, further studies are necessary to elucidate its role as a possible niche signal.

The intracellular role of CPE in pro-peptide processing and sorting is well documented [83], [85], but little is known about the autocrine/paracrine function of secreted CPE. Our *in* *vitro* neurosphere model uncovered a novel effect of secreted CPE. Treatment with the exogenous CPE resulted in fewer neurospheres and lower cell proliferation, suggesting that CPE can act as a negative regulator of NSC cell proliferation. This effect seems to be independent of the CPE’s carboxypeptidase activity because the culture medium we used was at physiological pH (∼7.4), which is not the optimal pH of 5.6 for the enzymatic activity of CPE in the secretory granules [83]. Such enzymatic-independent function of CPE could be achieved through receptor-mediated signaling. Based on the structural homology between the catalytic site within the amino-terminal domain of the Shh protein (Shh-N) and the catalytic site common to all carboxypeptidases, including CPE, it was speculated that CPE may work in a similar manner as a signaling molecule [89], but no experimental evidence has been reported. Alternatively, because CPE has a binding affinity for proteins other than pro-peptides [90], CPE may bind to selective proteins/peptides in the culture medium such as insulin or growth factors, thereby interfering with ligand-receptor interactions. However, the detailed underlying mechanism of the autocrine/paracrine function of CPE remains to be determined.

Although SST-REX is a useful method to screen secretory or membrane proteins, there exist some technical limitations including the limitation in the profile size. Because SST-REX is not a full-scale screening process at a saturated level, i.e., to screen the entire cDNA library, we could only test a limited number of the surviving factor-independent clones. Continued and repeated screening of the same library could increase the profile size. We initially identified a total of 307 cDNAs with the homology to known genes. However, we further limited our analysis to only 151 genes in our final SMEP to focus on only secretory and membrane associated molecules. Besides secretory and membrane associated molecules, approximately 40% of the initially identified genes encoded nonsecretory proteins. In agreement with our result, previous signal sequence trap studies have reported 25 to 50% false-positive rate, i.e., encoding proteins that are known to be not secreted [103]–[105]. One possible explanation for these false positives could be the transduced cDNAs that encode cell cycle regulatory proteins or cytoplasmic signaling molecules allowed IL-3-independent survival of Ba/F3 cells in our screen. For example, we identified cell cycle regulatory genes such as the cell division protein kinase 19 (*Cdk19*) and cytoplasmic signaling molecules such as mitogen-activated protein kinase 1 (*Mapk1*) in our SMEPs. Furthermore, the fact that subcellular organelle membrane proteins contain N-terminal hydrophobic residues, which might act as signal peptides [103], can further increase the false positive hits from the screening. Some of the expected molecules were not identified by our SST-REX (false-negative). For example, epidermal growth factor receptor (EGFR) protein is a type I transmembrane glycoprotein with the N-terminal signal sequence. EGFR is known for its expression in TAPs [61] but was not identified in our screening. Possible reasons for the false negatives include, 1) not present in the library due to primer or extension failure, 2) the expression of these proteins interferes Ba/F3 cell growth, 3) not screened at a saturated level [105].

For a systematic approach to identify NSC niche signals, various screening methods can be applied and each method has advantages and disadvantages. For example, SST-REX is useful to screen secretory or membrane proteins, but there are technical limitations as described above. Although cDNA microarrays aim for the genome-wide gene expression profiling, it can only detect genes that are already represented on the chip. Thus, combining data from different approaches will further improve the magnitude of gene expression profiling. Indeed, the identified genes in our NSC SMEP obtained by SST-REX did not overlap substantially with the potential NSC SMEP acquired from our microarray method, indicating that each approach could complement each other. Therefore, we employed a bioinformatics tool to refine the final SMEP and compared the cDNA microarray results as an alternative approach to widen the profile of NSCs for the secretory and cell-surface molecules. By combining SST-REX and microarray data, we identified several signaling molecules, which are known to modulate NSC behaviors. We found signaling pathway related molecules such as Shh receptor patched homolog 1 (*Ptch1*) [106], Wnt receptor frizzled homolog 2 (*Fzd2*) [107], Notch gene homolog 2 (*Notch2*) [108], and bone morphogenic protein receptor (*Bmpr1a*) [109] (Table S4 and Table S5). We also found interesting molecules that may be linked to NSC function, for example pleiotrophin (*Ptn*), midkine (*Mdk*), and protein tyrosine phosphatase receptor type Z polypeptide 1 (*Ptprz1*) (Table 1 and Table S5). Ptprz1 is a known receptor for Ptn and Mdk [110], [111] and it has been shown that they are involved in the growth of human embryonic stem cells [112], cerebellar Purkinje cell differentiation [113], and embryonic neural stem/progenitor cell survival/growth *in vitro* [114]. Since their potential roles in adult NSCs are not known, they can be candidate niche signaling molecules acting in an autocrine or paracrine manner.

To understand the nature of niche signals, it is important to consider how each niche cellular component contributes to the signaling network for maintaining the niche homeostasis. For this, it is indispensible to obtain the collective profile of each niche cell type. In this study, we report the first systematic approach to provide the SMEP of NSCs as well as other neighboring niche cells in the adult SVZ. Our approach helps to understand the molecular signature of the NSC niche and how niche signals can be orchestrated for the maintenance of homeostasis. It further demonstrates the importance of understanding the role of each niche cell type individually as well as in an integrated manner. Combining our niche cell isolation methods with genomics tools such as microarray or RNA-sequencing technologies will provide us further insights into the nature of NSC niche signals.

## Supporting Information

Table S1 Cell type-specific expression of marker genes.
(DOCX)

Table S2 Primer sequences used in qRT-PCR.
(DOCX)

Table S3 Secretory molecule expression profile (SMEP) data corresponding to published expression profiles.
(DOCX)

Table S4 Potential secretory molecule expression profile (SMEP) obtained by cDNA microarray. Secretory or transmembrane protein-encoding genes that were differentially expressed in SVZ NSCs and cerebellar Bergmann glia.
(DOCX)

Table S5 Potential secretory molecule expression profile (SMEP) obtained by cDNA microarray. Secretory or transmembrane protein-encoding genes that were most highly expressed in SVZ NSCs (top 2.5%).
(XLSX)
