# Supplementary material for: The Molecular Profiles of Neural Stem Cell Niche in the Adult Subventricular Zone
Source: PLoS One. 2012 Nov 29;7(11):e50501. doi: 10.1371/journal.pone.0050501 (PMC3510163; doi:10.1371/journal.pone.0050501)
Supplement: Table S1 — Cell type-specific expression of marker genes. (DOCX) [file pone.0050501.s001.docx]

**Table S1.** Cell type-specific expression of marker genes.

| **Cell type**  **Markers** | **NSC** | **TAP** | **Astrocyte** | **Ependymal cell** | **Endothelial cell** |
| --- | --- | --- | --- | --- | --- |
| **GFAP** | +++ | + | +++ | +/− |  |
| **Sox2** | +++ | +++ |  |  |  |
| **Nestin** | +++ | ++ |  |  |  |
| **Mash1** |  | +++ |  |  |  |
| **S100β** |  |  | ++ | +++ |  |
| **CD24** |  |  |  | ++ |  |
| **CD31** |  |  |  |  | +++ |
