# Supplementary material for: The Molecular Profiles of Neural Stem Cell Niche in the Adult Subventricular Zone
Source: PLoS One. 2012 Nov 29;7(11):e50501. doi: 10.1371/journal.pone.0050501 (PMC3510163; doi:10.1371/journal.pone.0050501)
Supplement: Table S2 — Primer sequences used in qRT-PCR. (DOCX) [file pone.0050501.s002.docx]

**Table S2.** Primer sequences used in qRT-PCR.

| **Gene** | **primer sequence (5' 🡺 3')** | |
| --- | --- | --- |
|  | **forward** | **reverse** |
| Validation of the SMEP | | |
|  |  |  |
| *Angpt2* | GAACCAGACAGCAGCACAAA | TCGAGTCTTGTCGTCTGGTTTA |
| *Apoe* | GACCCTGGAGGCTAAGGAC | CTGTCAGCAATGTGACCAACA |
| *Armet* | GAAAGACAGCCAGATCTGTGAAC | TTTCACCCGGAGCTTCTTCA |
| *Casq2* | GGATTGACCCAGATGACTTTCC | CCCAATCTGTGGCTTGAACA |
| *Cd34* | CCGAGCCATATGCTTACACA | ACCTCACTTCTCGGATTCCA |
| *Cd63* | CCTGCTGTCTGGTGAAGAGTA | TCCACTCCATGAAAAGACCAAAC |
| *CPE* | AAGTGGCAGTTCCTTTTAGCC | CCTCCTCCTTCCTTTCAGAGAA |
| *Cst3* | TACAGGTGGTGAGAGCTCGTA | GGCCCATCTCCACATCCAAA |
| *Efamp1* | TGTATGTGCCCACAGGGTTA | GCATTCATTGGTGGTCTCACA |
| *Emcn* | TTGCAACCACTCCATCAACC | TAACAACCAGCGCGATAACC |
| *Enpp2* | GGACGGACTGAAACAACTCA | GTCACATGTCACGTCTTCCA |
| *Folr1* | TGCAAGAGCAATTGGCACAA | CTCCCACAGGACACTCGTTA |
| *Htra1* | GATCCGAATGATGTCGCTCAC | CAGAGAGCACATCCGGGAA |
| *Igfbp2* | ACAAGCATGGCCGGTACAAC | GGTTCACACACCAGCACTCC |
| *Igfr1* | TTGGGCAATGGAGTGCTGTA | CTCCCATTCATCAGGCACGTA |
| *Kazald1* | GGAAGCTTCTGCAACCCTCA | TGATTGCAGCTGGGAGAATCC |
| *Layn* | CAGCCTGCCAGGACCTTTA | GGAAGGCTCATCCACATACCA |
| *Mesdc2* | AGCCTGTTCAACGCCAACTA | CCCATCCCGGAGCATGAA |
| *Nrcam* | GGACAGGAGTCCTTGGAATTCA | GTATGGTTCCACCCGGTCAA |
| *Ntrk2* | CACGGATGTTGCTGACCAAA | AGAATCCCACCACAGATGCA |
| *Prlr* | AGTAGATGGGGCCAGGAGAA | AGAACGGCCACAATGATCCA |
| *Ptgds* | GTGCAGCCCAACTTTCAACA | TACAGCTTTCTTCTCCCGGAAC |
| *Ptprz1* | AGCTCCAATCACCCAGACAA | TGTGTGAGCTTAACCCTGCTA |
| *Rarres2* | TGTGCAGTTGGCCTTCCAA | ACAAAGGTGCCAGCTGAGAA |
| *Scg3* | TACGGTACGATATCTCCAGAGGAA | GGTCTGCAGAGCAATTGTTTCA |
| *Sparcl1* | GTGTTTGCCAAGATCCAGAGAC | TGGCGTAGGTTTGGTTGTCA |
| *Tek* | GTTGGATGGCAATCGAATCAC | CCAGAGCAATACACCATAGGAC |
| *Ttr* | TGGACACCAAATCGTACTGGAA | AGTCGTTGGCTGTGAAAACC |
| *Vtn* | CGCATCAACTGTCAGGGGAA | TCCAGGACCCCATCCTCAAA |
| *Gapdh* | AGACGGCCGCATCTTCTT | TTCACACCGACCTTCACCAT |
|  |  |  |
| *in vitro* functional validation | | |
|  |  |  |
| *TuJ1* | TGGACAGTGTTCGGTCTGG | CCTCCGTATAGTGCCCTTTGG |
| *Gfap* | ATCCGCTCAGTCATCTTACCC | TGTCTGCTCAATGTCTTCCCTACC |
| *Olig2* | CACAGGAGGGACTGTGTCCT | GGTGCTGGAGGAAGATGACT |
| *Gapdh* | TGACATCAAGAAGGTGGTGAAGC | CCCTGTTGCTGTAGCCGTATTC |
|  |  |  |
| Validation of immunocytochemistry | | |
| *Gfap* | AGAACAACCTGGCTGCGTATA | CAGCGATTCAACCTTTCTCTCC |
| *Cd24* | GCAGATCTCCACTTACCGAAC | CAGTGCCAGAAGCAGCAA |
| *S100β* | ACAACGAGCTCTCTCACTTCC | ATCTTCGTCCAGCGTCTCC |
| *Gapdh* | AGACGGCCGCATCTTCTT | TTCACACCGACCTTCACCAT |
