# Supplementary material for: The Molecular Profiles of Neural Stem Cell Niche in the Adult Subventricular Zone
Source: PLoS One. 2012 Nov 29;7(11):e50501. doi: 10.1371/journal.pone.0050501 (PMC3510163; doi:10.1371/journal.pone.0050501)
Supplement: Table S3 — Secretory molecule expression profile (SMEP) data corresponding to published expression profiles. (DOCX) [file pone.0050501.s003.docx]

**Table S3.** Secretory molecule expression profile (SMEP) data corresponding to published expression profiles.

| **Profile** | **Gene symbol** | **Reference** |
| --- | --- | --- |
| NSC |  |  |
|  | *Cst3* | [1] |
|  | *Cnp* | [2] |
|  | *Apoe* | [3] |
|  | *Htra1* | [4] |
| TAP |  |  |
|  | *Cd63* | [5] |
|  | *Cd81* | [5] |
| Astrocyte |  |  |
|  | *Sparcl1* | [1] |
|  | *Apoe* | [1] |
|  | *Casq2* | [4] |
|  | *Scg3* | [5] |
|  | *Sparc* | [1] |
|  | *Timp4* | [4] |
|  | *Slc1a3* | [4] |
| Ependymal cell |  |  |
|  | *Apoe* | [6,7] |
|  | *Vtn* | [7] |
|  | *Ttr* | [4,6] |
| Choroid plexus |  |  |
|  | *Ttr* | [1] |
|  | *Clu* | [1] |
|  | *Cd81* | [8] |
|  | *Spint2* | [1] |
|  | *Folr1* | [1] |
|  | *Ptgds* | [8] |
|  | *Prlr* | [1] |
| Endothelial cell |  |  |
|  | *Cdh5* | [3] |
|  | *Cd34* | [9] |
|  | *Lamb1* | [9] |
|  | *Plxnd1* | [10] |
|  | *Lrp1* | [9] |
|  | *Vcam1* | [9] |
|  | *Cadm1* | [9] |
|  | *Cadm2* | [9] |
|  | *Cadm4* | [9] |
|  | *Tek* | [9] |
|  | *Itga1* | [9] |
|  | *Itga2b* | [9] |
|  | *Itgb1* | [9] |
|  | *Lama4* | [9] |
|  | *Lrp5* | [1] |
|  | *Vwf* | [9] |
| Neuroblast^[[1]](#footnote-0)^* | *Agrn* | [11] |
|  | *C3ar1* | [11] |
|  | *Ccl22* | [11] |
|  | *Ccl3* | [11] |
|  | *Ccl4* | [11] |
|  | *Cd24a* | [11] |
|  | *Cd86* | [11] |
|  | *Cdh11* | [11] |
|  | *Cdh2* | [11] |
|  | *Cxcl10* | [11] |
|  | *Cxcr7* | [11] |
|  | *Efnb1* | [11] |
|  | *Efnb2* | [11] |
|  | *Il1a* | [11] |
|  | *Il1b* | [11] |
|  | *Notch1* | [11] |
|  | *Npdc1* | [11] |
|  | *Plxna3* | [11] |
|  | *Sema4c* | [11] |
|  | *Slit1* | [11] |
|  | *St8sia2* | [11] |

**References (cited in Supporting Information, Table S2)**

1 Lein ES, Hawrylycz MJ, Ao N, Ayres M, Bensinger A, et al (2007) Genome-wide atlas of gene expression in the adult mouse brain. Nature 445: 168-176.

2 Karsten SL, Kudo LC, Jackson R, Sabatti C, Kornblum HI, Geschwind DH (2003) Global analysis of gene expression in neural progenitors reveals specific cell-cycle, signaling, and metabolic networks. Dev Biol 261: 165-182.

3 Tham M, Ramasamy S, Gan HT, Ramachandran A, Poonepalli A, et al (2010) CSPG is a secreted factor that stimulates neural stem cell survival possibly by enhanced EGFR signaling. PLoS One 5: e15341.

4 Beckervordersandforth R, Tripathi P, Ninkovic J, Bayam E, Lepier A, et al (2010) In vivo fate mapping and expression analysis reveals molecular hallmarks of prospectively isolated adult neural stem cells. Cell Stem Cell 7: 744-758.

5 Ahn JI, Lee KH, Shin DM, Shim JW, Kim CM, et al (2004) Temporal expression changes during differentiation of neural stem cells derived from mouse embryonic stem cell. J Cell Biochem 93: 563-578.

6 Jacquet BV, Salinas-Mondragon R, Liang H, Therit B, Buie JD, et al (2009) FoxJ1-dependent gene expression is required for differentiation of radial glia into ependymal cells and a subset of astrocytes in the postnatal brain. Development 136: 4021-4031.

7 Pfenninger CV, Steinhoff C, Hertwig F, Nuber UA (2011) Prospectively isolated CD133/CD24-positive ependymal cells from the adult spinal cord and lateral ventricle wall differ in their long-term in vitro self-renewal and in vivo gene expression. Glia 59: 68-81.

8 Marques F, Sousa JC, Coppola G, Gao F, Puga R, et al (2011) Transcriptome signature of the adult mouse choroid plexus. Fluids Barriers CNS 8: 10.

9 Chun HB, Scott M, Niessen S, Hoover H, Baird A, et al (2011) The proteome of mouse brain microvessel membranes and basal lamina. J Cereb Blood Flow Metab 31: 2267-2281.

10 Gong S, Zheng C, Doughty ML, Losos K, Didkovsky N, et al (2003) A gene expression atlas of the central nervous system based on bacterial artificial chromosomes. Nature 425: 917-925.

11 Pennartz S, Belvindrah R, Tomiuk S, Zimmer C, Hofmann K, et al (2004) Purification of neuronal precursors from the adult mouse brain: comprehensive gene expression analysis provides new insights into the control of cell migration, differentiation, and homeostasis. Mol Cell Neurosci 25: 692-706.

1. * Genes upregulated in PSA-NCAM+ cells compared to adult total brain were analyzed with DAVID to identify potential secretory or cell membrane associated proteins [11]. [↑](#footnote-ref-0)
