# Supplementary material for: The Molecular Profiles of Neural Stem Cell Niche in the Adult Subventricular Zone
Source: PLoS One. 2012 Nov 29;7(11):e50501. doi: 10.1371/journal.pone.0050501 (PMC3510163; doi:10.1371/journal.pone.0050501)
Supplement: Table S4 — Potential secretory molecule expression profile (SMEP) obtained by cDNA microarray. Secretory or transmembrane protein-encoding genes that were differentially expressed in SVZ NSCs and cerebellar Bergmann glia. (DOCX) [file pone.0050501.s004.docx]

**Table S4**. Potential secretory molecule expression profile (SMEP) obtained by cDNA microarray. Secretory or transmembrane protein-encoding genes that were differentially expressed in SVZ NSCs and cerebellar Bergmann glia.

| **Gene name** | **Gene symbol** | **Accession number** |
| --- | --- | --- |
|  |  |  |
| **up regulated in NSCs** |  |  |
| Fc receptor-like S, scavenger receptor | *Fcrls* | BC016551 |
| astrotactin 1 | *Astn1* | NM_007495 |
| Brevican | *Bcan* | BB335613 |
| carboxypeptidase E | *CPE* | BC010197 |
| cell adhesion molecule 1 | *Cadm1* | NM_018770 |
| colony stimulating factor 1 receptor | *Csf1r* | AI323359 |
| complement component 1, q subcomponent, beta polypeptide | *C1qb* | NM_009777 |
| delta/notch-like EGF-related receptor | *Dner* | AF370126 |
| disabled homolog 2 (Drosophila) | *Dab2* | AW986632 |
| endothelin converting enzyme 1 | *Ece1* | AW553715 |
| frizzled homolog 2 (Drosophila) | *Fzd2* | BB371406 |
| glutamate receptor, ionotropic, AMPA2 (alpha 2) | *Gria2* | NM_013540 |
| glutamate receptor, ionotropic, NMDA2C (epsilon 3) | *Grin2c* | NM_010350 |
| glypican 5 | *Gpc5* | BB171986 |
| hyaluronan and proteoglycan link protein 1 | *Hapln1* | AF098460 |
| insulin-like growth factor binding protein 5 | *Igfbp5* | NM_010518 |
| leucine-rich repeat LGI family, member 1 | *Lgi1* | AV332442 |
| mannose receptor, C type 1 | *Mrc1* | NM_008625 |
| netrin 4 | *Ntn4* | BQ086474 |
| neurexin I | *Nrxn1* | BB336165 |
| neurocan | *Ncan* | BB804298 |
| neuronal guanine nucleotide exchange factor | *Ngef* | NM_019867 |
| platelet factor 4 | *Pf4* | NM_019932 |
| progestin and adipoQ receptor family member VII | *Paqr7* | BC022922 |
| serglycin | *Srgn* | NM_011157 |
| solute carrier family 15 (H+/peptide transporter), member 2 | *Slc15a2* | NM_021301 |
| solute carrier family 6 member 11 | *Slc6a11* | BB536871 |
| sortilin-related VPS10 domain containing receptor 2 | *Sorcs2* | NM_030889 |
| thymus cell antigen 1, theta | *Thy1* | NM_009382 |
| trophoblast glycoprotein | *Tpbg* | BQ177165 |
|  |  |  |
| **down regulated in NSCs** |  |  |
| EMI domain containing 1 | *Emid1* | NM_080595 |
| 1-acylglycerol-3-phosphate O-acyltransferase 4 | *Agpat4* | BE994529 |
| 2',3'-cyclic nucleotide 3' phosphodiesterase | *Cnp* | BB251922 |
| alpha-2-macroglobulin | *A2m* | NM_175628.3 |
| ankyrin 2, brain | *Ank2* | BB136541 |
| ankyrin repeat domain 43 | *Ankrd43* | BB428991 |
| anoctamin 6 | *Ano6* | BG074632 |
| calcium channel, voltage-dependent, gamma subunit 5 | *Cacng5* | BQ174680 |
| calcium channel, voltage-dependent, gamma subunit 7 | *Cacng7* | AF361349 |
| cell adhesion molecule with homology to L1CAM | *Chl1* | NM_007697 |
| chloride channel 3 | *Clcn3* | BI739053 |
| collagen triple helix repeat containing 1 | *Cthrc1* | AK003674 |
| collagen, type II, alpha 1 | *Col2a1* | NM_031163 |
| cortexin 3 | *Ctxn3* | BM116248 |
| delta/notch-like EGF-related receptor | *Dner* | AF370126 |
| ecotropic viral integration site 2a | *Evi2a* | NM_010161 |
| ephrin B2 | *Efnb2* | U30244 |
| family with sequence similarity 173, member A | *Fam173a* | BC008155 |
| fibromodulin | *Fmod* | BB532202 |
| frizzled homolog 1 (Drosophila) | *Fzd1* | NM_021457 |
| gamma-aminobutyric acid (GABA) A receptor, subunit alpha 4 | *Gabra4* | NM_010251 |
| gap junction membrane channel protein epsilon 1 | *Gjc3* | NM_080450 |
| glutamate receptor, ionotropic, AMPA1 (alpha 1) | *Gria1* | NM_008165 |
| glutamate receptor, ionotropic, AMPA4 (alpha 4) | *Gria4* | NM_019691 |
| glutamate receptor, ionotropic, delta 2 | *Grid2* | NM_008167 |
| glycoprotein m6b | *Gpm6b* | AF254879 |
| growth arrest specific 1 | *Gas1* | BB550400 |
| growth differentiation factor 10 | *Gdf10* | L42114 |
| HtrA serine peptidase 3 | *Htra3* | NM_030127 |
| kit ligand | *Kitl* | NM_013598 |
| Kv channel interacting protein 3, calsenilin | *Kcnip3* | AF300870 |
| latrophilin 3 | *Lphn3* | NM_198702 |
| membrane protein, palmitoylated 6 | *Mpp6* | AF199010 |
| meteorin, glial cell differentiation regulator | *Metrn* | NM_133719 |
| NEL-like 2 (chicken) | *Nell2* | AI838010 |
| neurofascin | *Nfasc* | NM_182716 |
| neuropeptide Y | *Npy* | NM_023456 |
| progestin and adipoQ receptor family member VIII | *Paqr8* | AV328983 |
| protein tyrosine phosphatase, receptor type, D | *Ptprd* | NM_011211 |
| protocadherin 9 | *Pcdh9* | BQ177394 |
| S100 calcium binding protein A10 (calpactin) | *S100a10* | NM_009112 |
| selenoprotein | *Sep15* | NM_053102 |
| signal sequence receptor, beta | *Ssr2* | NM_025448 |
| similar to cadherin 22; cadherin 22 | *Cdh22* | NM_174988 |
| solute carrier family 14 (urea transporter), member 1 | *Slc14a1* | AW556396 |
| solute carrier family 22 (organic cation transporter), member 4 | *Slc22a4* | BC010590 |
| solute carrier family 38, member 1 | *Slc38a1* | BF165681 |
| solute carrier organic anion transporter family, member 4a1 | *Slco4a1* | AV348121 |
| tensin 3 | *Tns3* | AI315031 |
| tetraspanin 6 | *Tspan6* | NM_019656 |
| thioesterase superfamily member 4 | *Them4* | BC022612 |
| tissue inhibitor of metalloproteinase 4 | *Timp4* | NM_080639 |
